# Functional comparisons of the virus sensor RIG-I from humans, the microbat *Myotis daubentonii*, and the megabat *Rousettus aegyptiacus*, and their response to SARS-CoV-2 infection

**DOI:** 10.1128/jvi.00205-23

**Published:** 2023-09-20

**Authors:** Andreas Schoen, Martin Hölzer, Marcel A. Müller, Kai B. Wallerang, Christian Drosten, Manja Marz, Benjamin Lamp, Friedemann Weber

**Affiliations:** 1 Institute for Virology, FB10-Veterinary Medicine, Justus-Liebig University, Giessen, Germany; 2 RNA Bioinformatics and High-Throughput Analysis, Friedrich Schiller University Jena, Jena, Germany; 3 European Virus Bioinformatics Center, Jena, Germany; 4 German Centre for Infection Research (DZIF), Partner Sites Giessen and Charité, Berlin, Germany; 5 Institute of Virology, Charité-Universitätsmedizin Berlin, corporate member of Freie Universität Berlin, Humboldt-Universität zu Berlin, and Berlin Institute of Health, Berlin, Germany; The Peter Doherty Institute for Infection and Immunity, Melbourne, Victoria, Australia

**Keywords:** bat, *Chiroptera*, interferon system, retinoic acid-inducible gene-I, RIG-I, *Myotis daubentonii*, *Yangochiroptera*, *Rousettus aegyptiacus*, *Yinpterochiroptera*, SARS-CoV-2

## Abstract

Bats (order *Chiroptera*) are a major reservoir for emerging and re-emerging zoonotic viruses. Their tolerance toward highly pathogenic human viruses led to the hypothesis that bats may possess an especially active antiviral interferon (IFN) system. Here, we cloned and functionally characterized the virus RNA sensor, retinoic acid-inducible gene-I (RIG-I), from the “microbat” *Myotis daubentonii* (suborder *Yangochiroptera*) and the “megabat” *Rousettus aegyptiacus* (suborder Yinpterochiroptera) and compared them to the human ortholog. Our data show that the overall sequence and domain organization are highly conserved and that all three RIG-I orthologs can mediate a similar IFN induction in response to viral RNA at 37° and 39°C but not at 30°C. Like human RIG-I, bat RIG-Is were optimally activated by double stranded RNA containing a 5’-triphosphate end and required mitochondrial antiviral-signaling protein (MAVS) for antiviral signaling. Moreover, the RIG-I orthologs of humans and of *R. aegyptiacus*, but not of *M. daubentonii*, enable innate immune sensing of SARS-CoV-2 infection. Our results thus show that microbats and megabats express a RIG-I that is not substantially different from the human counterpart with respect to function, temperature dependency, antiviral signaling, and RNA ligand properties, and that human and megabat RIG-I are able to sense SARS-CoV-2 infection.

**IMPORTANCE:**

A common hypothesis holds that bats (order *Chiroptera*) are outstanding reservoirs for zoonotic viruses because of a special antiviral interferon (IFN) system. However, functional studies about key components of the bat IFN system are rare. RIG-I is a cellular sensor for viral RNA signatures that activates the antiviral signaling chain to induce IFN. We cloned and functionally characterized RIG-I genes from two species of the suborders *Yangochiroptera* and *Yinpterochiroptera*. The bat RIG-Is were conserved in their sequence and domain organization, and similar to human RIG-I in (i) mediating virus- and IFN-activated gene expression, (ii) antiviral signaling, (iii) temperature dependence, and (iv) recognition of RNA ligands. Moreover, RIG-I of *Rousettus aegyptiacus* (suborder *Yinpterochiroptera*) and of humans were found to recognize SARS-CoV-2 infection. Thus, members of both bat suborders encode RIG-Is that are comparable to their human counterpart. The ability of bats to harbor zoonotic viruses therefore seems due to other features.

## INTRODUCTION

Bats (order *Chiroptera*), along with rodents, are assumed to be the most important reservoirs of zoonotic viruses (1 – 5). Several of the bat-borne pathogens (mostly RNA viruses) can cause severe disease in humans, e.g., SARS coronaviruses 1 and 2 (5 – 7) or Marburg viruses (8). The taxonomic order *Chiroptera* was recently divided into the two suborders *Yangochiroptera* and *Yinpterochiroptera* (9), which largely, but not entirely, overlap with the previous division into microbats (having the ability of echolocation) and megabats (large fruit eating bats), respectively. Although members of both suborders can host highly pathogenic viruses, they rarely show clinical signs of disease, indicating an ability to tolerate and resist infection to an unprecedented level (10). Infection tolerance is proposed to be mediated by a dampening of pro-inflammatory responses that would otherwise lead to tissue damage (11 – 15). Infection resistance is supposed to be due to special features of the antiviral type I interferon (IFN) system, which is in bats under strong positive selection (16 – 20). In line with this, some *Yinpterochiroptera* megabats exhibit elevated base levels of type I IFNs (21, 22) or IFN-stimulated genes (ISGs) (23), whereas for others this was not the case but an expanded tissue distribution of the master IFN regulator IRF7, diversified IFN gene loci, or a set of nonstandard ISGs were detected (24 – 27). *Myotis* “microbats” (*Yangochiroptera*) express uniquely high paralog numbers of the broadly antiviral IFN effectors Tetherin (BST2) and PKR (28 – 30).

Type I IFNs (IFN-α/β) are cytokines that constitute the first line of defense against viral infection (31). Several intra- and extracellular pattern recognition receptors (PRRs) are able to sense viral hallmark structures called pathogen-associated molecular patterns (PAMPs), e.g., genomic RNA (32), and initiate a signal transduction chain that leads to the upregulation of IFN genes, first of all IFN-β. The RNA sensor retinoic acid inducible gene I (RIG-I) is one of the most important PRRs for virus infection (33, 34). It has three major domains: an N-terminal domain encompassing two caspase recruitment domains (CARDs), a central DExD/H box RNA helicase domain, and a C-terminal domain involved in ligand binding and regulation (35, 36). RIG-I recognizes double-stranded RNA (dsRNA) structures—especially when containing a 5’-triphosphate moiety—as it is present in many RNA virus genomes (37 – 40). RNA ligand-bound RIG-I then undergoes a conformational change to expose the N-terminal CARD domains, thus enabling interaction with the signal adaptor mitochondrial antiviral signaling protein (MAVS) and the eventual activation of the IFN transcription factor, interferon regulatory factor 3 (IRF3) (34). Newly produced and secreted IFN then binds to its cognate receptor on the cell surface to activate the expression of antivirally active ISGs, thus establishing a virus-resistant cellular state (31).

So far, knowledge on RIG-I genes of bats is mostly limited to nucleotide sequences, sequence comparisons, genomic organization, and expression (41 – 46). The known bat RIG-I amino acid sequences (which are all bar one from *Yinpterochiroptera* megabats) exhibited approximately 70% to 90% similarity to RIG-I of other mammals, suggesting a conserved domain organization (43, 45). Basal mRNA levels of bat RIG-I were elevated in immune associated tissues, e.g., spleen, and could be stimulated by virus infection, the dsRNA mimetic poly I:C (41 – 43, 45 – 47), and type IFN (29, 48).

However, despite the importance of bats as major hosts for zoonotic viruses and the central role of RIG in antiviral defense, functional data on bat RIG-I proteins are entirely lacking. To fill this gap, we cloned and expressed RIG-I sequences from two bat species from “microbats” and “megabats”, namely *Myotis daubentonii* (belonging to the *Yangochiroptera*) and *Rousettus aegyptiacus* (belonging to the *Yinpterochiroptera*), investigated their function in virus recognition, temperature dependence, PAMP binding and IFN induction, and compared them to human RIG-I. Moreover, we tested the involvement of the bat RIG-Is in recognition of SARS-CoV-2 infection.

## RESULTS

### Bat cells induce an innate immune response upon virus infection

**Fig 1:**
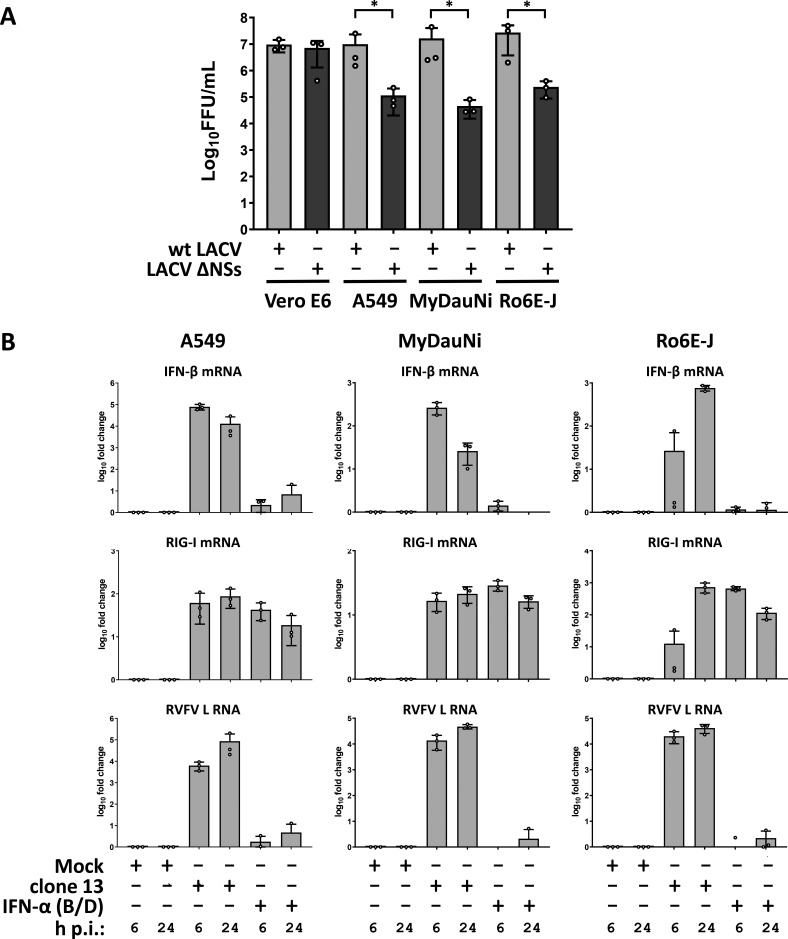
Type I Interferon competence of micro- and megabat cells lines. (**A**) African green monkey kidney cells (Vero E6), human A549, *M. daubentonii* (MyDauNi) and *R. aegyptiacus* (Ro6E-J) cells were infected with wt LACV or the IFN-sensitive LACVΔNSs (MOI 0.01). After 72 hours, the supernatants were collected and the viral titers determined. The graphs show log_10_ titers, mean values and standard deviations from three independent replicates. **(B**) A549, MyDauNi, and Ro6E-J cells were either mock treated, infected with RVFV clone 13 (MOI 5), or treated with pan-species IFN-α (B/D) (1,000 U/mL) for 6 or 24 hours, respectively. Expression of IFN-β, RIG-I, and RVFV L RNAs was monitored by RT-qPCR. The minimal amounts of RVFV L RNA that were detected in uninfected cells were most likely due to spillover. The graphs show data points for log_10_ induction over mock, with mean values and standard deviations from three independent replicates. *P < 0.05

We have previously shown that cell lines derived from *M. daubentonii* (MyDaNi) and *R. aegyptiacus* (Ro6E-J) are capable of responding to exogenously added pan-species IFN (29, 49). Here, we investigated whether these cells could also produce endogenous antiviral IFN in response to virus infection. We employed a pair of genetically matched viruses that are either inducing IFN (La Crosse virus lacking the IFN suppressor NSs; LACVΔNSs), or blocking IFN induction (La Crosse virus expressing the IFN suppressor NSs; wt LACV) (50). Replication of the NSs-deleted virus mutant LACVΔNSs is severely reduced in IFN competent cells and animals (51). Besides the two bat cell lines, we employed simian Vero E6 cells, which lack type I IFN expression (52) as a negative control, and the IFN competent human A549 cells as a positive control. Each of the cell lines was infected with either of the two viruses at a low MOI (0.01) to ensure multistep growth, and incubated for 72 hours. Figure 1A shows that supernatants harvested from Vero E6 cells contained similar amounts of infectious viruses, indicating comparable replication. In the cell lines A549, MyDauNi and Ro6E-J, by contrast, yields of the LACV ΔNSs were reduced by approximately two orders of magnitude compared to the wt LACV. Thus, just as in the human positive control line A549, the two bat cell lines are restricting replication of the IFN-sensitive virus mutant, strongly suggesting that the bat cell lines have mounted an antiviral IFN response.

To directly compare how the bat cells activate genes for endogenous IFN and ISGs (including RIG-I), we performed RT-qPCR analyses for RIG-I (DDX58) as well as for a series of other antiviral marker genes. IFN-β thereby represents exclusively virus-dependent genes (29, 53), similar to the chemokine CXCL10 (54) which is however also inducible by IFN-γ (55). Mx1 (MxA in humans) is established as an ISG that only reacts to IFN, but not directly to infection (56), whereas OAS1 can be induced by both virus infection and IFN (53). The A549, MyDaNi, and Ro6E-J cells were either infected with the strong IFN-inducing Rift Valley fever virus (RVFV) mutant clone 13 (54) or treated with 1,000 U/mL pan-species IFN-α. Total RNA samples were taken 6 and 24 hours later and tested for mRNA levels of the mentioned marker genes using RT-qPCR. Upon virus infection, all three cell lines induced IFN-β as expected but with different kinetics (Fig. 1B). A549 and MyDauNi cells showed an initial peak at 6 hours post infection (p.i.), which then decreased to the later 24 hours p.i. time point while Ro6E-J cells had a delayed IFN-β induction which peaked 24 hours p.i. (Fig. 1B). Levels of viral RNA were however similar between the two bat cell lines, indicating true differences in IFN induction kinetics. RIG-I was upregulated in all three cell lines by clone 13 infection and by IFN-α treatment, but again the Ro6E-J cells reacted not as quickly to infection as the other two cell lines (see Fig. 1B). A similar Ro6E-J-specific pattern was observed for CXCL10, whereas all cells upregulated Mx1 and OAS1 in a similar manner (Fig. S1). These results indicate that the applied micro- and megabat cells are fully competent in launching an antiviral IFN response to viral infection or type I IFN treatment.

### Amino acid sequence comparison of bat RIG-Is

**Fig 2:**
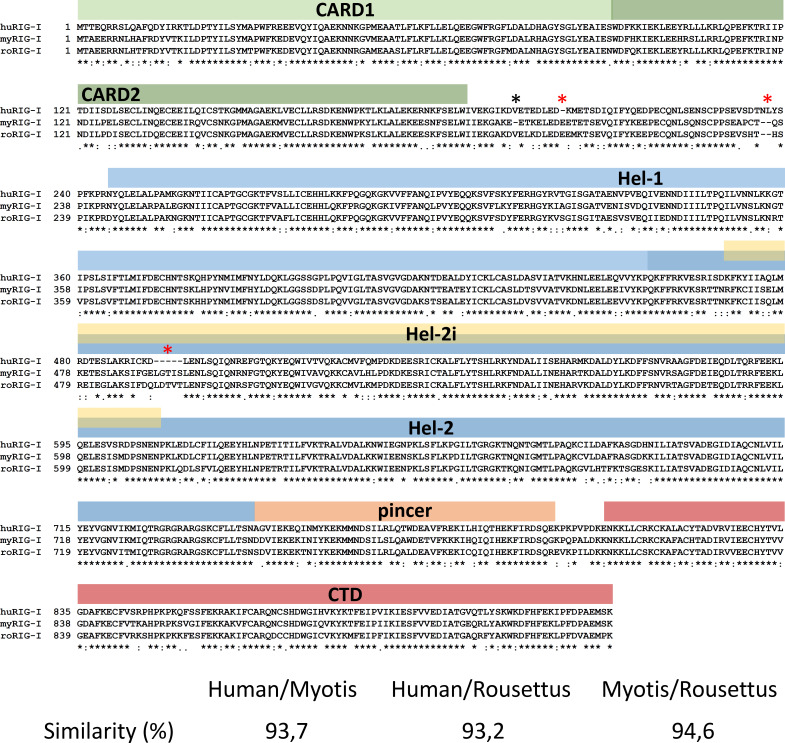
Amino acid sequence alignment for human, micro- and megabat RIG-I. Identical amino acid residues are marked with a small asterisk (*), replacements with conserved (:), semi-conserved residues with dots (.), gaps with a dash (-). Above the alignment is the different domains of human RIG-I depicted based on Kowalinski et al. (35). RIG-I sequences derived from human, *M. daubentonii*, and *R. aegyptiacus* are designated as huRIG-I, myRIG-I, and roRIG-I, respectively. The indels distinguishing human and bat RIG-I sequences are marked by big red asterisks, and the *Myotis*-specific deletion is marked by a big black asterisk.

As a first step towards functional characterization of bat RIG-I orthologs, we assembled the full-length RIG-I sequence from *M. daubentonii*, using the data from our transcriptome studies (29, 49). Figure 2 shows the amino acid sequences alignment of the human, *M. daubentonii* and *R. aegyptiacus* RIG-I. Overall the sequences are well conserved, with 93.2% to 94.6% similarity between the different species. However, both bat orthologs exhibit three apparent differences to human RIG-I (red asterisks in the figure), namely an insertion of one amino acid and a deletion of two amino acids in the hinge region between the CARDs and the helicase domain, and an insertion of five amino acids in the Hel-2i helicase subdomain which is responsible for auto-inhibition of RIG-I in the absence of an RNA ligand (35). Moreover, *M. daubentonii* RIG-I has a specific one-amino acid deletion in the hinge region (black asterisks in the figure). An extended multiple alignment that includes RIG-I amino acid sequences from 15 additional bat species confirmed the deletions and insertions identified with *M. daubentonii* and *R. aegyptiacus* RIG-I for the other members of their respective suborders (including the *Yangochiroptera*-specific one-amino acid deletion in the hinge region), with the only exception that *Pipistrellus kuhlii* species (*Yangochiroptera*) lacks the five amino acids insertion in the Hel-2i helicase subdomain (Fig. S2).

Using the SMART database (http://smart.embl-heidelberg.de/), we identified the two N-terminal CARDs followed by a DEXD-like helicase domain, a helicase domain and the C-terminal regulatory domain as being conserved between all three RIG-Is (Fig. S3). Thus, while the overall sequence and domain organization are highly conserved, the bat RIG-Is contain several specific indels.

### Bat RIG-Is are functional

**Fig 3:**
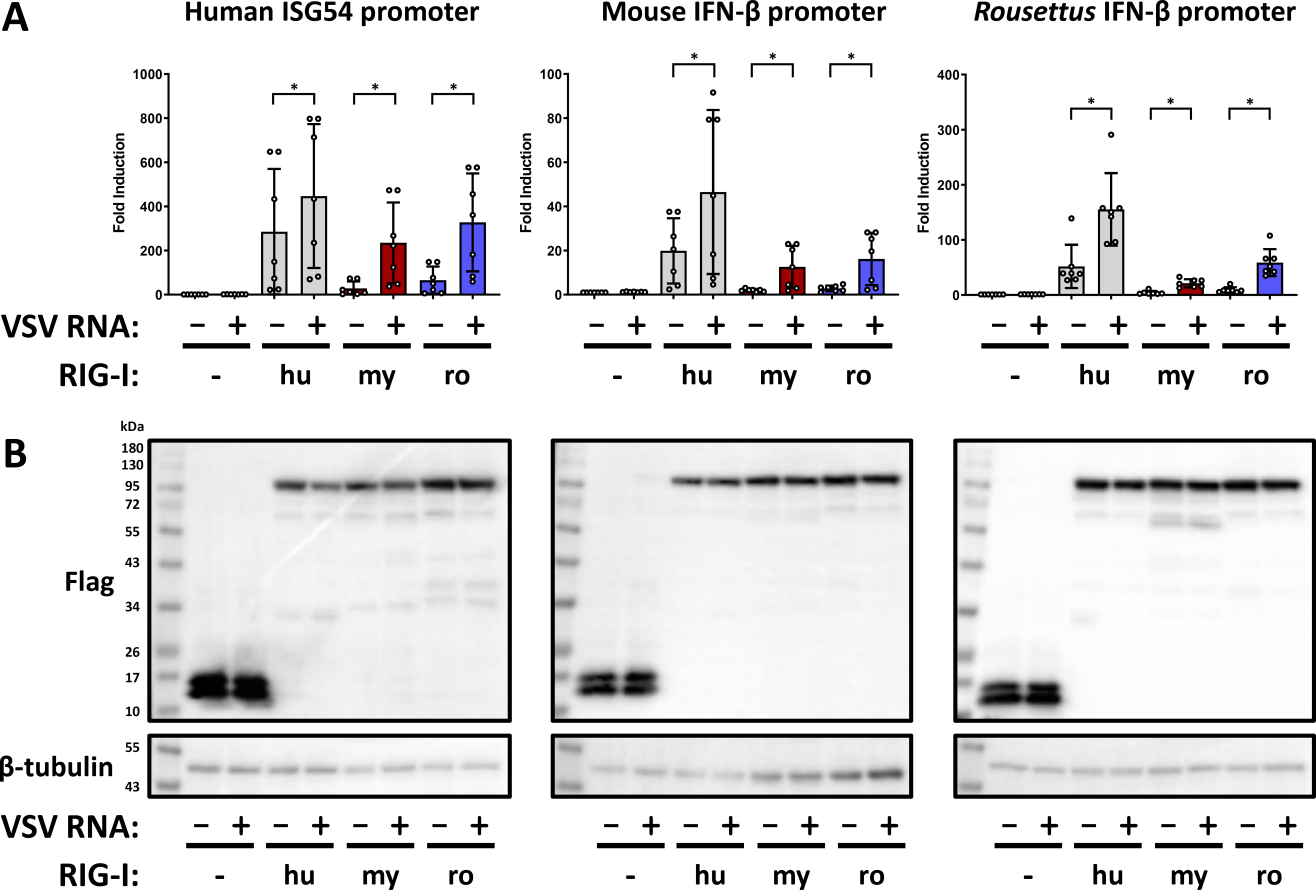
Transcomplementation of RIG-I-deficient human cells by bat RIG-I orthologs. (**A**) HEK293 ΔRIG-I cells were transfected with plasmids encoding 3xFlag-ΔMx control protein (-), or 3xFlag-tagged hu-, my-, or roRIG-I together with firefly-luciferase expressing plasmids under the control of the indicated innate immune promoters and *Renilla*-luciferase under SV40-control promoter. After 24 hours, the cells were stimulated with VSV genomic RNA for 16 hours. Upon harvesting the cells, the firefly/*Renilla* luciferase activities were measured. The promoter activation by human RIG-I in the absence of VSV RNA is most likely due to the fact that cells were incubated for 40 hours after transfection of the RIG-I expression construct. Graphs show data points for fold induction over the untreated negative control (column 1), with mean values and standard deviations from seven independent replicates. (**B**) Immunoblot analysis was performed with antibodies against the indicated antigens. Representative data from five independent experiments are shown. *P < 0.05.

Attempts to knock down endogenous RIG-I in the bat cells or to find antibodies with sufficient cross-reactivity were unsuccessful (data not shown). We, therefore, constructed cDNA plasmids to express the three RIG-I orthologs, each equipped with an N-terminal 3×Flag epitope, in RIG-I-deficient cells. Then, we used reporter assays to test whether human HEK293 ΔRIG-I cells (39) could be transcomplemented with the cloned bat orthologs. The cells were transfected with the different RIG-I constructs [or a 3×Flag-control (CTRL) protein] together with plasmids encoding firefly luciferase under the control of the inducible human ISG54-promoter and a transfection control plasmids encoding *Renilla* luciferase under the control of the SV40-promoter. Since the ISG54 promoter is inducible by both virus infection and IFN, we also employed the exclusively virus-responsive IFN-β-promoters of mouse and of *R. aegyptiacus* in parallel. At 24 hours after plasmid transfections, the inducible promoters were stimulated by supertransfection of the cells with the RIG-activating genome RNA of vesicular stomatitis virus (VSV). Following a further 16 hours of incubation, firefly (inducible promoter) and *Renilla* (transfection baseline for normalization) luciferase activities were measured. The normalized data in Fig. 3A show an absence of inducible firefly luciferase activity in the CTRL-transfected samples, as expected, whereas expression of the human RIG-I alone already resulted in a 30–400 fold induction, depending on the promoter. As the CTRL-transfected cells did also not respond after VSV RNA transfection, any specific activity detected when RIG-Is are overexpressed is due to transcomplementation. When the cells expressing human RIG-I had been stimulated with VSV RNA, overall induction levels were 1.5- to 2.5-fold higher than in the unstimulated counterpart (see Fig. 3A). Unlike human RIG-I, the bat RIG-I orthologs did not exhibit background promoter induction, but in presence of VSV RNA they stimulated the promoters by 20- to 300-fold for *Myotis* and 20- to 400-fold for *Rousettus* (see Fig. 3A).

To make sure that the differences in induction levels were not due to differences in RIG-I expression, we analyzed the cell lysates by immunoblotting. All three RIG-Is from human, *Myotis* and *Rousettus* showed comparable levels and the expected apparent molecular weight of approximately 100 kDa (Fig. 3B). Moreover, we could also successfully transcomplement RIG-I^–/–^ mouse embryo fibroblasts with all three RIG-I orthologs, indicating that the RIG-I of humans and bats can rescue RIG-I deficiency irrespective of the species background (Fig. S4).

Although the promoter activations exhibited a wide variation which is most likely owed to the fact that the HEK293 ΔRIG-I cells (which are also quite sensitive) had to be transfected twice, these results demonstrate that the RIG-I orthologs cloned from micro- and megabat cells can be stimulated by viral RNA and are able to initiate an antiviral signaling that results in the transactivation of virus-responsive promoters.

### Bat and human RIG-I show a similar induction pattern under different temperatures

**Fig 4:**
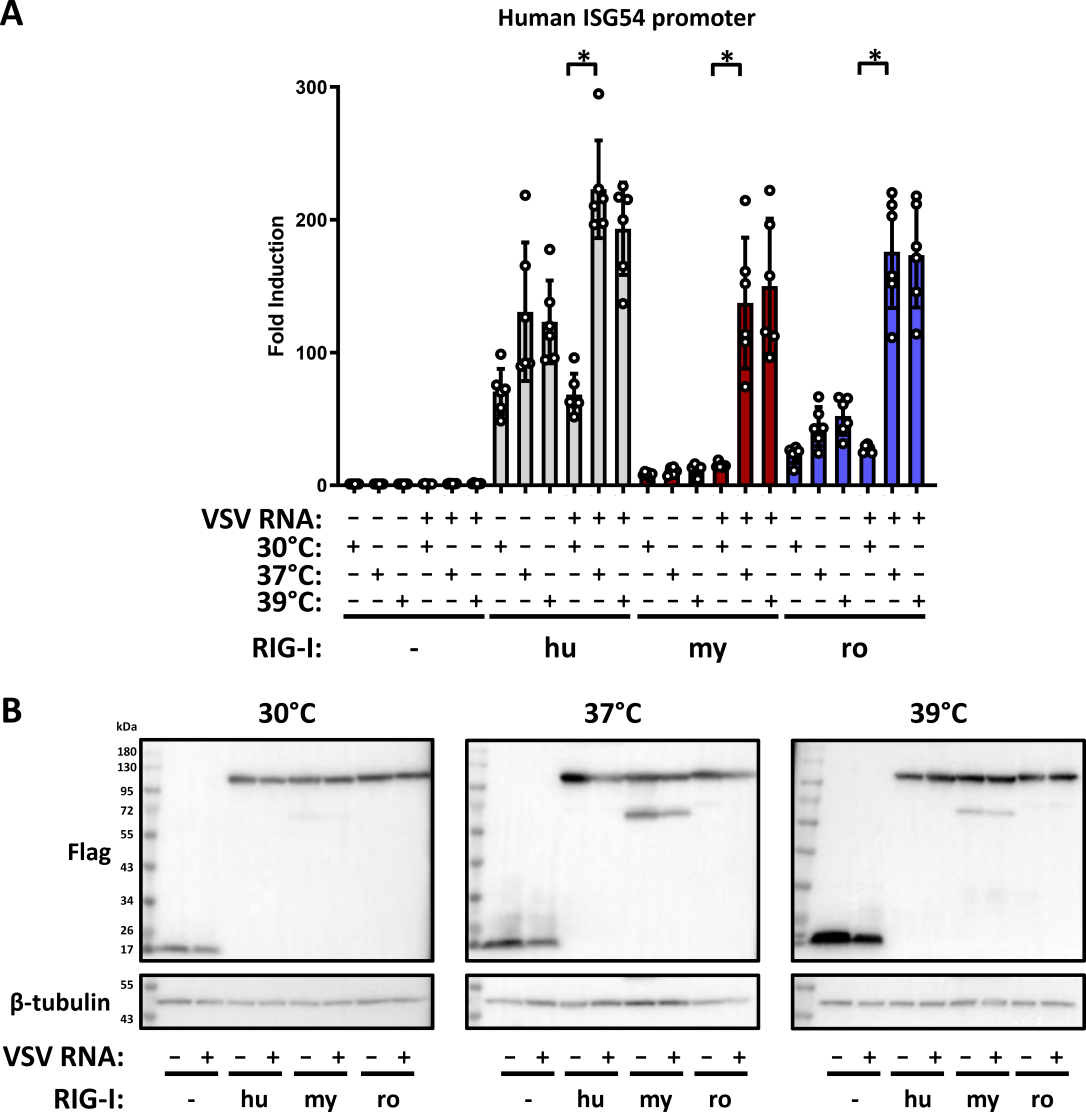
Temperature dependent IFN induction by exogenously expressed human and bat RIG-I orthologs. (**A**) HEK293 ΔRIG-I cells were transfected, stimulated and assayed as described for Fig. 3, but incubated at different temperatures as indicated. Each graph shows the results from six independent replicates. (**B**) Immunoblot analysis was performed with antibodies against the indicated antigens. Representative data from three independent experiments are shown. *P < 0.05.

The body temperature of bats is remarkably variable, from down to 11°C during sleep or torpor (e.g., in hibernation) to up to 41°C during flight (57). As the IFN system is known to be influenced by temperature (58), we wondered whether temperature might affect the activity of the bat RIG-I orthologs. To test this, we transcomplemented the HEK293 ΔRIG-I cells and allowed RIG-I expression for 24 hours at 37°C, but then placed the cells in incubators set to 30°C, 37°C, or 39°C at 1 hour before stimulation with VSV RNA, and kept them for another 16 hours at the different temperatures (the cells did not tolerate 41°C, data not shown). As shown in Fig. 4A, at 30°C none of the RIG-I orthologs could be specifically stimulated by VSV RNA. At 37°C, there was a robust activation as shown above, which was maintained at the temperature of 39°C. RIG-I expression levels were comparable at all temperatures, with an additional smaller band observed for *Myotis* RIG-I at 37°C and 39°C (Fig. 4B). The appearance of the band might indicate RIG-I degradation, enforced at higher temperatures. Taken together, these results suggest that the human and bat RIG-Is are functional at normal body temperature or higher but not at 30°C.

### Bat RIG-I signaling via MAVS

**Fig 5:**
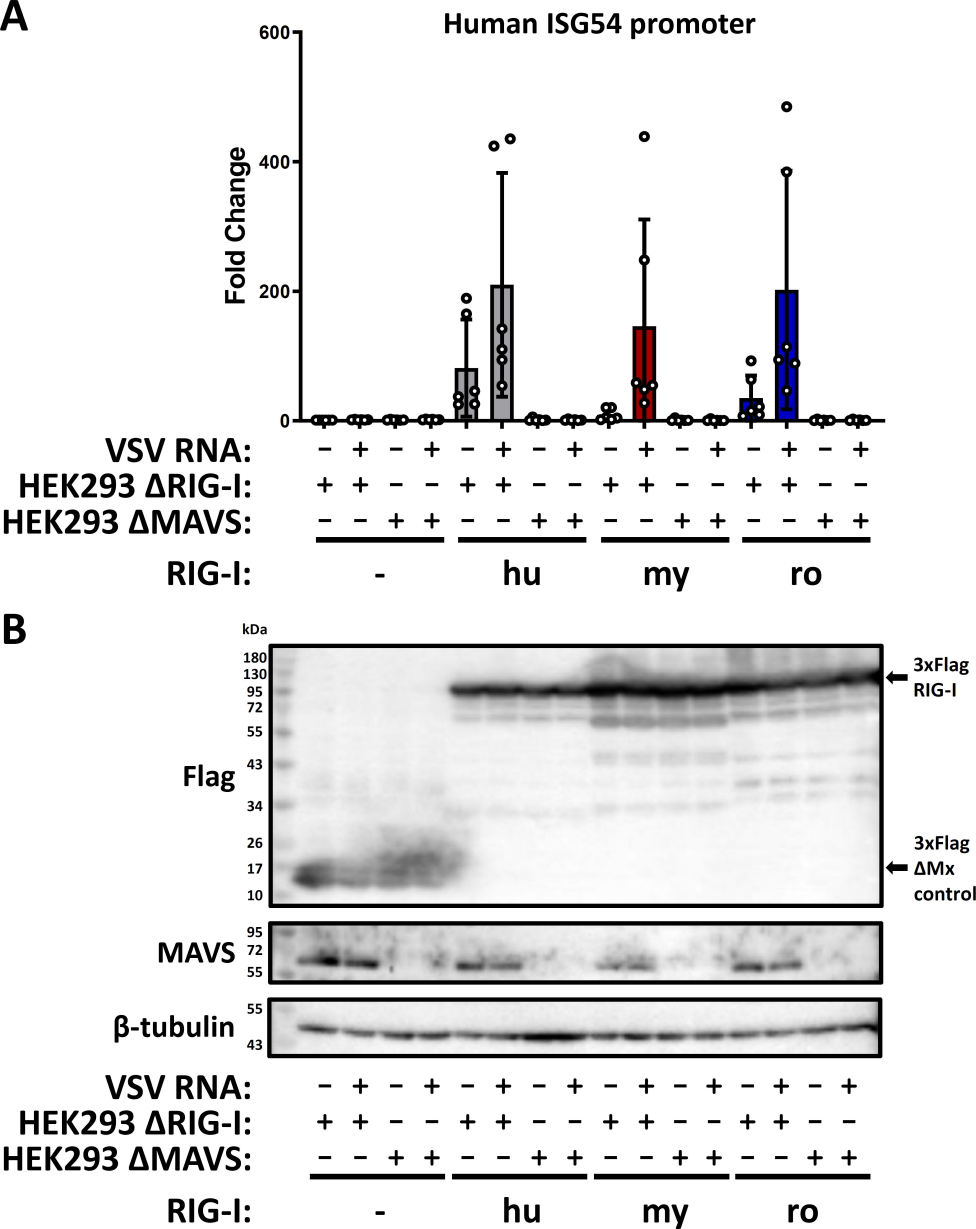
MAVS-dependent antiviral signaling by human and bat RIG-I orthologs. (**A**) HEK293 ΔRIG-I or ΔMAVS cells were transfected, stimulated, and assayed as described in Fig. 3. The graph shows the results from six independent replicates. (**B**) Immunoblot analysis was performed with antibodies against the indicated antigens. The expected size of the 3xFlag-ΔMx control (−) and RIG-I proteins are indicated with arrows. Representative data from five independent experiments are shown.

Upon recognition of 5′-triphosphorylated dsRNA, RIG-I undergoes a conformational change to expose the CARDs for interaction with MAVS which, in turn, initiates the antiviral signaling. To investigate whether the bat RIG-I orthologs are also signaling via MAVS, we compared their ability to activate the ISG54-promoter reporter in HEK293 ΔRIG-I and HEK293 ΔMAVS cells. The cells with the respective genotype were transfected with the expression constructs and stimulated with VSV RNA as described. RIG-I-dependent ISG54-promoter activation was only detected in the presence of MAVS (Fig. 5A), with comparable expression levels of the Flag-tagged RIG-I orthologs (Fig. 5B). Thus, the bat RIG-Is, like the human ortholog, are signaling via MAVS.

### RNA ligand interaction

**Fig 6:**
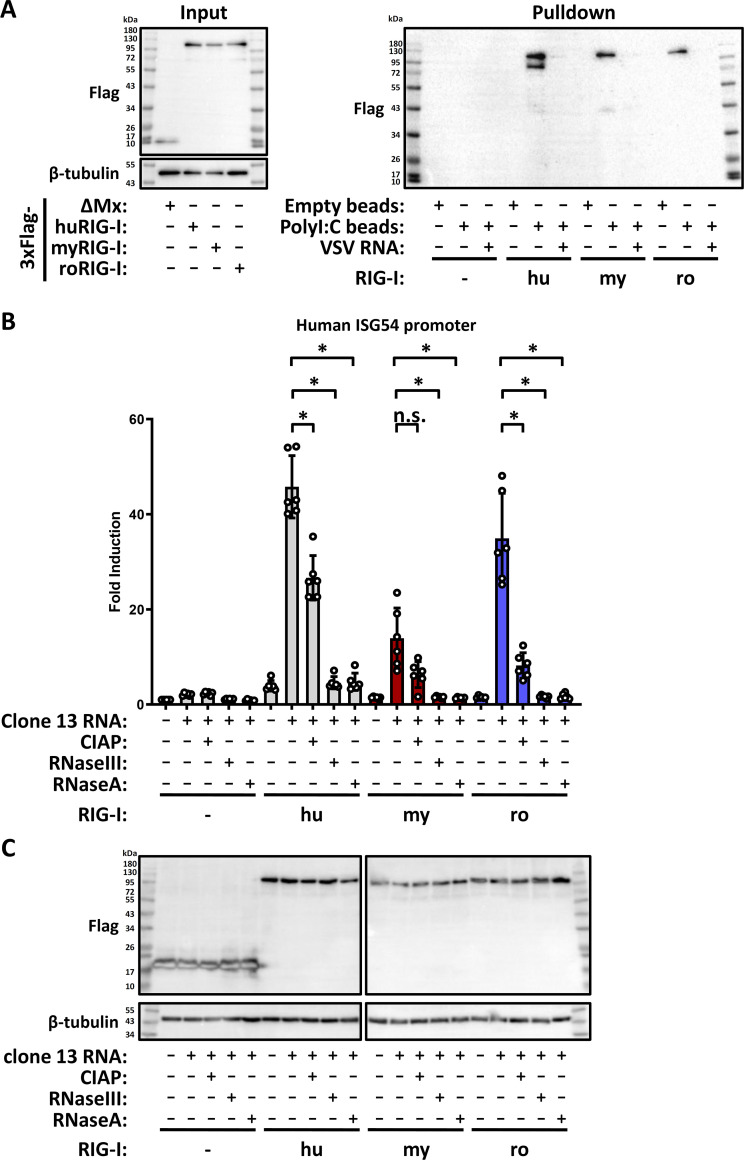
Involvement of double stranded RNA and 5’-phosphorylation in RIG-I activation. (**A**) PolyI:C pulldown. Cell lysates from HEK293 ΔRIG-I cells transfected with plasmids encoding 3xFlag-ΔMx, hu-, my-, or roRIG-I were incubated with magnetic beads coupled, or not, with HMW-polyI:C. Possible binding of the respective RIG-I to the polyI:C was competed with free VSV genomic RNA. After incubation and elution, immunoblot analysis was performed for the Flag epitope tag. Representative data from three independent experiments are shown. (**B**) HEK293 ΔRIG-I cells were transfected with cDNA constructs for 3xFlag-ΔMx, hu-, my-, or roRIG-I and stimulated with VSV genomic RNA that was pretreated with the different enzymes as indicated. After 24 hours, the cells were harvested and the firefly/*Renilla* luciferase activity was measured. The graph shows the results from six independent replicates. (**C**) Immunoblot analysis using antibodies against the indicated antigens. Representative data from six independent experiments are shown. n.s., non-significant; *P < 0.05.

We investigated the interaction of the bat RIG-I orthologs with RNA. Firstly, we performed pulldowns with the biotin-labeled dsRNA analog polyI:C (HMW) that is bound to streptavidin-beads. Cell lysates from HEK293 ΔRIG-I cells expressing either Flag-tagged control or RIG-I proteins (Fig. 6A, left panel) were mixed with the polyI:C-coated beads. To make sure that the binding was specific, we also incubated the lysates with either empty beads or added free VSV RNA as a competitor. After incubation, washing, and elution, the eluates were subjected to SDS-PAGE and immunoblot detection of the Flag epitope tag (Fig. 6A, right panel). None of the RIG-I orthologs bound unspecifically to the empty beads, but all exhibited a binding to the poly I:C-coated beads which could be efficiently outcompeted by free VSV RNA.

Next, we investigated whether the activation of the RIG-I orthologs depends on the 5′-triphosphate moiety and the double-strandedness of a viral RNA. To this aim, genomic RNA isolated from RVFV clone 13 particles were either mock, Calf Intestinal Alkaline Phosphatase (CIAP, 5′-phosphatase), RNaseIII (double-stranded RNA (dsRNA) specific endoribonuclease), or RNaseA treated. The RNAs were then transfected into HEK293 ΔRIG-I cells that had been transfected with the RIG-I expressing plasmids and the ISG54-reporter plasmid. After 24 hours of incubation, ISG54 promoter activity was measured. Pretreatment of the RNA with CIAP more than halved the induction by all RIG-Is compared to mock treated RNA and pretreatment with either of the two RNases reduced the induction level to background, irrespective of the particular RIG-I (Fig. 6B and C). Together with the polyI:C pulldown, the above results indicate that bat RIG-I, like human and mouse RIG-I (32, 34), are specifically binding dsRNA and that full activation by viral genome RNA requires both a 5′-triphosphate and double stranded RNA.

### IFN induction by SARS-CoV-2 can be enabled by RIG-I orthologs

**Fig 7:**
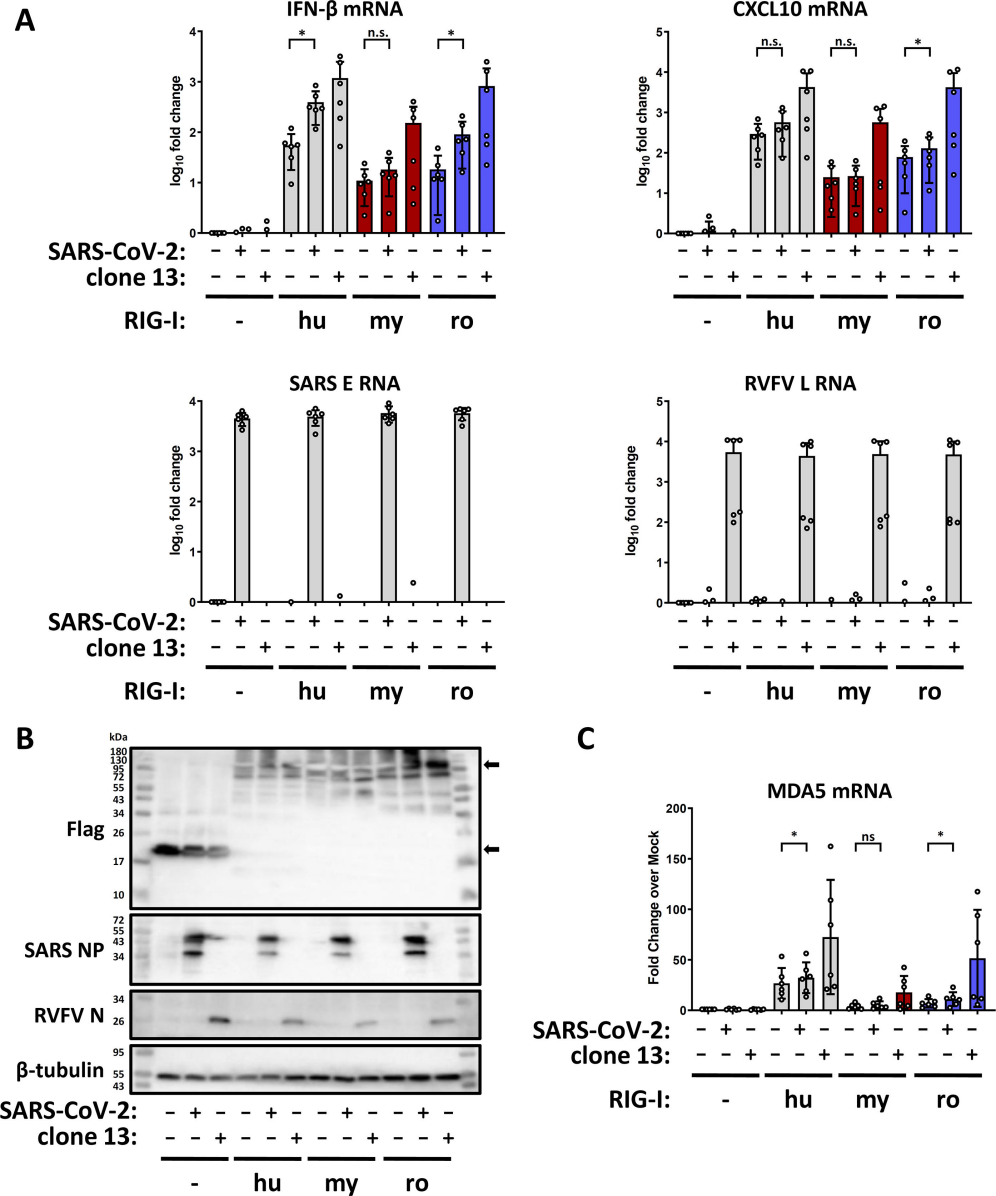
Innate immune response to SARS-CoV-2 in cells overexpressing RIG-I orthologs. (**A**) ACE2-HEK293 ΔRIG-I cells were transfected with the indicated plasmids for 24 hours, followed by infection with either SARS-CoV-2 or RVFV clone 13 (MOI 1). After 16 hours, total RNA was isolated and used for detection of IFN-β and CXCL10 mRNA, SARS E RNA and RVFV L RNA by RT-qPCR. The graph shows the results from six independent replicates. (**B**) Immunoblot analysis with antibodies against the indicated antigens. Representative data from six independent experiments are shown. (**C**) RT-qPCR analysis for MDA5 mRNA with the samples used in (A). ns, non-significant; *P < 0.05.

*Rhinolophus* megabats (suborder *Yinpterochiroptera*) are proposed to be reservoirs of SARS-coronaviruses including the pandemic SARS-CoV-2 (5). Infection of human lung epithelial cells with SARS-CoV-2 induces a certain level of type I IFN (59), which was mostly found to be mediated by MDA5, a PRR that is structurally and functionally related to RIG-I (60 – 62). However, also RIG-I was found to be involved in IFN and cytokine induction by SARS-CoV-2, either directly via antiviral signaling (63, 64), or indirectly by controlling viral RNA synthesis down to non-inducing levels (65). We tested the involvement of our bat RIG-I orthologs for their ability to sense SARS-CoV-2 infection. Transcomplementation experiments in human ACE2-HEK293 ΔRIG-I cells demonstrated that the RIG-I orthologs of humans and of *R. aegyptiacus* are indeed capable of inducing IFN-β mRNA synthesis in response to SARS-CoV-2 (Fig. 7A, top left panel). For the RIG-I of *M. daubentonii*, by contrast, the reaction to SARS-CoV-2 was not statistically significant. *R. aegyptiacus* RIG-I also raised mRNA levels of the chemokine CXCL10, but just by a factor below 2, whereas for the RIG-Is from the two other species CXCL10 induction was not statistically significant (Fig. 7A, top right panel). For both human and *R. aegyptiacus* RIG-I orthologs, the cytokine mRNA induction levels by SARS-CoV-2 were approximately five to more than tenfold lower than for the positive control virus clone 13. This is expected since clone 13 is a RVFV mutant devoid of any IFN antagonistic factor (66, 67), whereas SARS-CoV-2 is a wild-type virus expressing several proteins that counteract IFN induction (68, 69). No differences in SARS or RVFV RNA (Fig. 7A, bottom panels) or protein levels (Fig. 7B) were observed between the negative control (3×Flag-ΔMx) and the RIG-I expressing samples, and expression of all three RIG-I orthologs was confirmed (Fig. 7B; Fig. S5). Thus, the RIG-I orthologs of both humans and the megabat *R. aegyptiacus* enable innate immune sensing of SARS-CoV-2, whereas for the RIG-I of the microbat *M. daubentonii* we could not measure a statistically significant effect. However, expression of the various RIG-I orthologs also induced MDA5 (Fig. 7C). Although the levels of MDA5 induction were comparatively low, overexpressing RIG-I orthologs could not entirely clarify whether the IFN response to SARS-CoV-2 was owed to the respective RIG-I orthologs, or rather to the endogenous MDA5 they are inducing.

### MDA5 is dispensable for SARS-CoV-2 innate immune sensing

**Fig 8:**
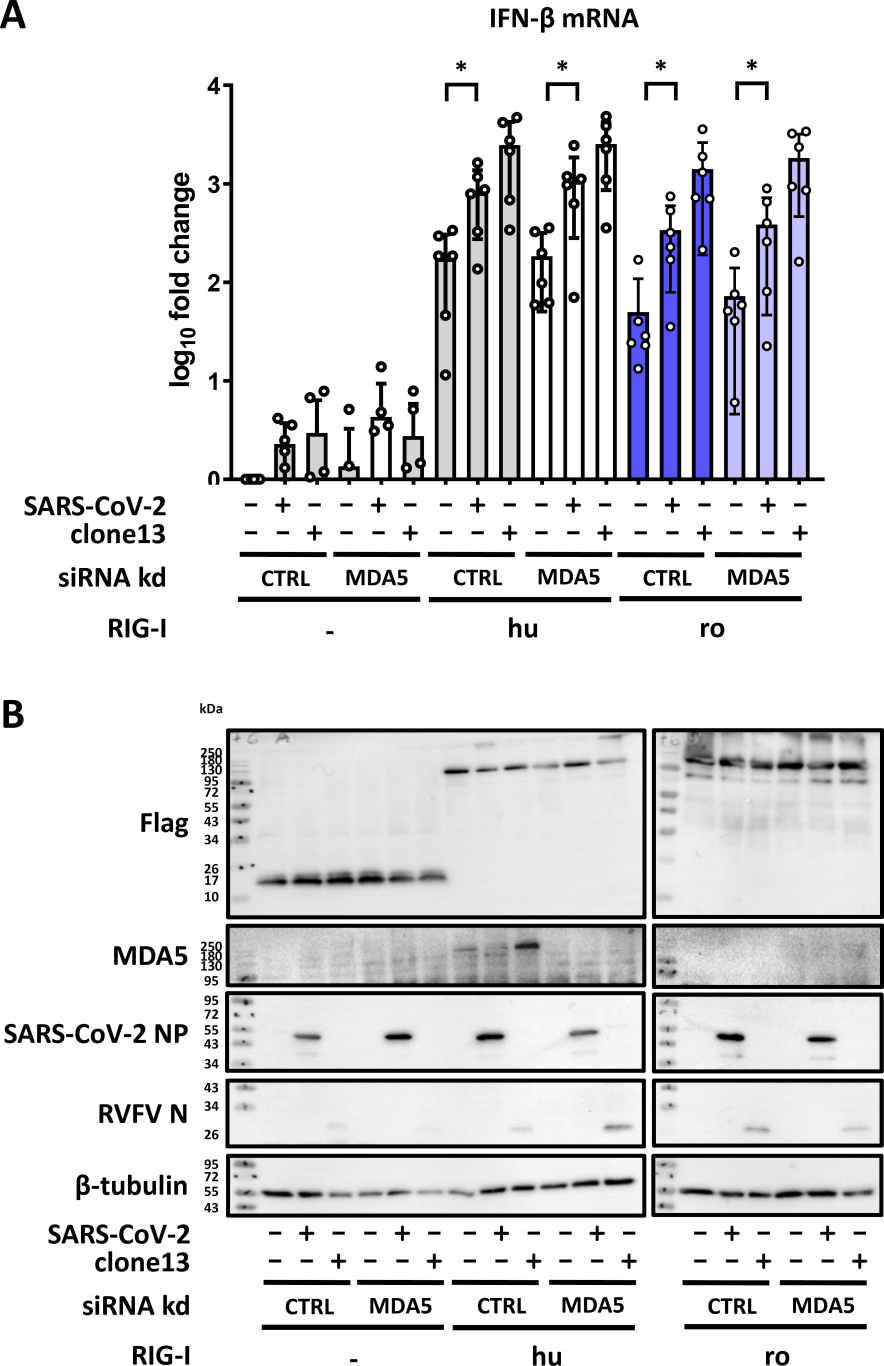
Innate immune response to SARS-CoV-2 is RIG-I dependent. (**A**) Expression of endogenous MDA5 was siRNA-knocked down in the ACE2-HEK293 ΔRIG-I cells. The cells were then transfected with the indicated plasmids for 24 hours, followed by infection with either SARS-CoV-2 or RVFV clone 13 (MOI 1). After 16 hours, total RNA was isolated and used for detection of IFN-β mRNA by RT-qPCR. The graph shows the results from six independent replicates. (**B**) Immunoblot analysis with antibodies against the indicated antigens. Endogenous MDA5 protein could only be detected under conditions of huRIG-I overexpression, most likely because the bat RIG-Is have lower basal activity. Representative data from six independent experiments are shown. *P < 0.05.

To investigate whether the RIG-I-dependent IFN induction by SARS-CoV-2 could be indirectly mediated, we abrogated MDA5 expression by siRNA treatment before transfection of ACE2-HEK293 ΔRIG-I cells with the two SARS-CoV-2-reactive RIG-I orthologs (human and *R. aegyptiacus*). As shown in Fig. 8A, the IFN induction in response to SARS-CoV-2 occurred irrespective of whether cells were treated with the control siRNA or with the MDA5 siRNA, as long as human or *R. aegyptiacus* RIG-I were present. The previously seen (low) effect of SARS-CoV-2 on CXCL10 induction could not be reproduced in the siRNA-transfected cells (Fig. S6A). Again, even in the RIG-I expressing cells the MDA5 levels were comparatively low and suppressed by the specific siRNA as expected (Fig. 8B; Fig. S6B through D). Moreover, viral RNA levels including those of SARS-CoV-2 were not influenced by the overexpressed RIG-I orthologs (Fig. S6E and F). Thus, in our system MDA5 seems not to contribute to the RIG-I-mediated IFN induction by SARS-CoV-2.

Overall, these experiments demonstrate that the RIG-I orthologs of *R. aegyptiacus* and humans, but not of *M. daubentonii*, are indeed capable of sensing infection by SARS-CoV-2.

## DISCUSSION

RIG-I is responsible for one of the earliest steps of the innate immune response, the recognition of intruding viral RNA and the induction of antiviral IFNs (34, 40). Since bats are a major reservoir for zoonotic viruses and suspected to have a special IFN system (1, 3, 5), we set out to deep-characterize RIG-I orthologs from one species each of the microbats (*M. daubentonii*, suborder *Yangochiroptera*) and the megabats (*R. aegyptiacus*, suborder *Yinpterochiroptera*), and compare them to human RIG-I.

In terms of gene expression, we could stimulate mRNA synthesis of the bat RIG-I orthologs in parental cells by type I IFN treatment as well as by virus infection, results that are in line with previous findings (29, 41 – 46, 48). The amino acid sequences derived from cloned RIG-I cDNAs were more than 90% similar to each other and compared to human RIG-I (with the notable exception of small indels) and the predicted domain structure (34) was obviously conserved.

Our functional characterization of the *M. daubentonii* and *R. aegyptiacus* RIG-I orthologs suggest that bat RIG-Is are not substantially different from the human counterpart. All three RIG-I orthologs were capable of binding dsRNA, are in their function partially dependent on the 5’-triphosphate RNA end, are signaling via MAVS, and can trigger the induction of IFN and other cytokines in response to viral RNA or virus infection. In the overexpression experiments, neither protein levels nor dsRNA binding or 5′-triphosphate end dependency were substantially different. Nonetheless, the RIG-I of *M. daubentonii*, exhibited in most settings a somewhat reduced activity, whereas *R. aegyptiacus* RIG-I tended more often to perform comparably to human RIG-I. Importantly, the reduced activity by *M. daubentonii* RIG-I occurred in both a human and a mouse cell background. When parental bat cells were stimulated with a RIG-I-inducing virus, by contrast, neither the antiviral response in total nor IFN induction *per se* was much different between the *M. daubentonii* and *R. aegyptiacus* RIG-I. Thus, *Myotis* RIG-I may not possess a systematically lower antiviral signaling capability, but rather need a cofactor that is only present or only fitting in parental cells and regulates a signaling step downstream of RNA recognition. The most obvious possibility would be MAVS as the prime interactor and downstream antiviral signal transmitter. Further studies are however necessary to clarify this.

As RIG-I is a key PRR for the very first step of the IFN response (34), it is often targeted by viral counterstrategies (68 – 71). Some highly pathogenic viruses modify their 5′ genome ends to avoid recognition by RIG-I (72, 73), whereas others encode IFN antagonists that directly interact (74 – 79) or induce its degradation (80, 81). Also the bat-borne Nipah virus as well as SARS-CoV-2 express proteins with such activities (78, 82, 83). Moreover, it is known that species-specific differences in the matching of viral IFN antagonists to their cellular targets correlate with host range or virulence (84, 85). It will therefore be interesting to see whether and to what extent the *Myotis* and *Rousettus* RIG-Is we characterized as well as human RIG-I can be inhibited by antagonists from bat viruses, and whether such interactions take place in conserved domains that are also present on human RIG-I. Testing viral RIG-I antagonists for potential broad-species IFN antagonism may help to assess the zoonosis risk of newly identified bat viruses.

The involvement of RIG-I in IFN induction by SARS-CoV-2 is still not entirely clarified. While most reports concluded that MDA5 rather than RIG-I is the relevant PRR (60 – 62), two studies reported it is additionally RIG-I-dependent (63, 64). Moreover, a direct antiviral effect of RIG-I against SARS-CoV-2 was shown (65), and the RIG-I antagonistic activity of SARS-CoV-2 proteins also indicates a certain relevance for the virus (82, 83). In our hands, both human and *R. aegyptiacus* RIG-I enabled HEK293 ΔRIG-I cells to induce IFN in response to SARS-CoV-2 infection and in an MDA5-independent manner. RIG-I, therefore, seems to be another PRR sensing SARS-CoV-2 under certain conditions, which is in line with the results by Arai et al. who identified RIG-I-activating small virus RNAs produced excessively by SARS-CoV-2, but not by the common cold coronaviruses OC43 and 229E (63). Besides this, it is interesting that the RIG-I of *R. aegyptiacus*, but not of *M. daubentonii,* is reacting to SARS-CoV-2 as *Rousettus* belongs to the same suborder *Yinpterochiroptera* as *Rhinolophus*, the major host of SARS-coronaviruses (5).

Unlike humans, bats have to endure extreme changes in body temperature when they switch between torpor, sleep, and flight (57, 86). Torpor is a state of reduced metabolic activity, applied, e.g., during hibernation (87, 88). Despite the principal differences between humans and bats regarding temperature regulation, we did not observe any species-specific activity of the respective RIG-Is that would have been temperature dependent. The two bat and the human RIG-I orthologs showed RNA-responsive activity at 37°C and at 39°C, while at 30°C there was only background activity (which was highest for the human ortholog). We cannot exclude a dominant influence of the human cell background, i.e., that cellular factors of the antiviral signaling chain other than RIG-I are shutting down at lower temperatures. Our observations, however, are in agreement with studies showing a reduced and delayed IFN system activity at lower temperatures in human, primate, and mouse systems (58) and the lack of any transcriptional response in virus-infected *Myotis myotis* cells at conditions simulating torpor (89). Our data showing the absence of RIG-I activity at low temperature are also compatible with the report that hibernating bats elicit very little immune responses to a fungal pathogen (86). Taken together, there appears to be a relative immune dormancy at low body temperature, which is expected to render the animals more susceptible to virus infections. Therefore, torpor, which also occurs in tropical bats as a response to environmental heat (87), may contribute to the role of bats as virus reservoir.

The antiviral IFN system of bats has attracted great attention as it might be the key to understand the role of these animals as virus reservoirs. The results presented are corroborating previous studies and predictions on the high similarity of bat RIG-I orthologs with those of other mammals regarding sequence, domain structure, and regulation. Our functional data on dsRNA binding and the dependency on 5′-triphosphate ends, MAVS, and temperature, as well as on the recognition of SARS-CoV-2 infection do not indicate substantial differences between the RIG-Is of humans and bats. This insight complements recent observations that the antiviral IFN effectors Tetherin and PKR of bats are basically functioning the same way as their human counterparts (28, 30). On the other hand, however, compared to humans some bats encode multiple copies of the genes for Tetherin and PKR as well as for IFNs or express non-standard ISGs (24 – 26, 28 – 30). Thus, it could be speculated that the IFN system of bats has functionally conserved key factors but is probably enforced by increased copy numbers of antiviral genes or novel ISGs.

In any case, it is hoped that the data and tools we generated will enable further insights into the role of the antiviral IFN system for the unique capacity of bats to harbor a huge range of different human pathogenic viruses without falling prey to them.

## MATERIALS AND METHODS

### Cells and viruses

A549, MyDauNi (MyDauNi/2 c), Vero E6, BHK, HEK293 ΔRIG-I or ΔMAVS, ACE2-HEK293 ΔRIG-I, MEF RIG-I−/− were maintained in DMEM supplemented with 17.8 mg/L L-alanine, 0.7 g/L glycine, 75 mg/L L-glutamic acid, 25 mg/L L-proline, 0.1 mg/L biotin, 25 mg/L hypoxanthine, 3.7 g/L sodium bicarbonate, 10% fetal calf serum (FCS), 2 mM glutamine, 120 U/mL penicillin, and 100 g/mL streptomycin while Ro6E-J were cultivated in DMEM (Gibco, 21969035) supplemented with 10% fetal calf serum (FCS), 2 mM glutamine, 120 U/mL penicillin, 100 g/mL streptomycin (Gibco, 10378016), and non-essential amino acids (Gibco, 11140050). Stocks of RVFV clone 13, LACV wt, LACV ΔNSs, and VSV were grown on BHK cells while SARS-CoV-2 [München-1.2/2020/984 (B.1) (90)] was propagated on VeroE6 cells, infected with an MOI of 0.001, for 72 (RVFV/LACV/SARS-CoV-2) or 48 hours for VSV. The respective stock was then titrated on Vero E6 for SARS-CoV-2, using an overlay medium containing MEM (Gibco, 21935028) supplemented with 10% fetal bovine serum, 2 mM glutamine, 120 U/mL penicillin,100 g/mL streptomycin (Gibco, 10378016). and 1,5% Avicell (91). RVFV clone 13, LACV wt/ΔNSs, and SARS-CoV-2 were incubated for 72 hours while VSV was incubated for 24 hours. The overlay was then removed and the cells fixed and stained using staining solution (0.75% crystal violet, 3.75% formaldehyde, 20% ethanol, and 1% methanol).

### RT-qPCR analyses

The cells were infected as indicated in the legends to Fig. 1, 7 and 8; Fig. S4. After the indicted time point, total cellular RNA was isolated using RNeasy Mini Kit (Qiagen, Cat No./ID: 74106) according to manufacturer’s instructions. A total of 100 ng isolated RNA was used for cDNA synthesis using PrimeScript High Fidelity RT-PCR Kit (Takara, R022B). RT-qPCR was performed using TB Green Premix Ex Taq (Tli RNase H Plus) (Takara, RR420B) according to manufacturer’s instructions on an Applied biosystems StepOnePlus machine. For detection of human transcripts, QuantiTect Primer Assay (Qiagen) against 18S ribosomal RNA (QT00199367), IFN-β (QT00203763), RIG-I (QT00040509), CXCL10 (QT01003065), MxA (QT00090895), OAS1 (QT00099134), and MDA5 (QT00033789) were used. For the detection of full-length transcript sequences for *M. daubentonii*, we used an available *de novo* transcriptome assembly based on bulk RNA-Seq data (SRR8062281-SRR8062299) from our previous study (29). In short, we applied an ensemble approach as described in (92) combining the output of different transcriptome assembly tools and used the final assembly (available at https://osf.io/x9kad) for detecting full-length transcripts for *M. daubentonii*. For *R. aegyptiacus*, we used publicly available genome and annotation data to obtain transcript sequences (GCF_014176215.1). Primers were designed using Primer3Plus (https://primer3plus.com/cgi-bin/dev/primer3plus.cgi) and ordered from Eurofins. For detection of *M. daubentonii* 18S ribosomal RNA: fwd 5′ AAACGGCTACCACATCCAAG 3′ and rev 5′ CCTCCAATGGATCCTCGTTA 3′, IFN-β: fwd 5′ AAAGCAGCAATTCAGCCTGT 3′ and rev 5′ CTGCTGGAGCATCTCGTACA 3′, RIG-I: fwd 5′ GGAAAACCACAACCTGCACT 3′ and rev 5′ ACTCTTTGGTCTGGGGTGTG 3′, CXCL10: fwd 5′ TTTTCTGCCTCATCCTTCTGA 3′’ and rev 5′ TGGACAAGATGGACTTGCAG 3′, IFN-λ3: fwd 5′ CACATCCACTCCAAGCTTCA 3′ and rev 5′ TCAGCGACACATCTCAGGTC 3′, Mx1: fwd 5′ CAGAGGGAGAGGGCTTTCTT 3′ and rev 5′ TCTGCTGGTTCTCCTTTATTTG 3′, and OAS1: fwd 5′ AGCCATTGACACCATCTGCA 3′ and rev 5′ CTCTTGCTGACATGCTTCCA 3′’ while for *R. aegyptiacus* 18S ribosomal RNA: fwd 5′ CGCGGTTCTATTTTGTTGGT 3′ and rev 5′ AGTCGGCATCGTTTATGGTC 3′, IFN-β: fwd 5′ ATTGCCTCAAGGACAGGATG 3′ and rev 5′ TTCAGTTTCTCCAGGGCTGT 3′, RIG-I: fwd 5′ CAAAAGCACAAGTGAAGCCT 3′, and rev 5′ TTGTCGGTAGTCCGTGATTC 3′, CXCL10: fwd 5′ TCAACCTGTTAATCCAAAGTCC 3′ and rev 5′ CCTTTCCTTGCTAATTGCTTTC 3′, IFN-λ3: fwd 5′ ACCTCCACCACTGGCTGT 3′ and rev 5′ AATGGCAACACGTTTCAGGT 3′, Mx1: fwd 5′ TCGGCTGTTTACCAAAATCC 3′ and rev 5′ CCAGGGTTTTGATTTGCTGT 3′ and OAS1: fwd 5′ CTATGCTTGGGAACGTGGAT 3′, and rev 5′ GGCCAACTCTGTGAGTCTCC 3′ were used. RVFV L segment RNA and SARS E RNA were detected with PrimeDirect Probe RT-qPCR Mix (Takara, RR650A) according to manufacturer’s instructions using RVFV L primers fwd 5′ TGAAAATTCCTGAGACACATGG 3′, rev 5′ ACTTCCTTGCATCATCTGATG 3′ and probe 5′ 6FAM-CAATGTAAGGGGCCTGTGTGGACTTGTG-BHQ1 3′ (93) or the SARS-CoV-2 E primers fwd 5′ ACAGGTACGTTAATAGTTAATAGCGT 3′, rev 5′ ATATTGCAGCAGTACGCACACA 3′ and probe 5′ FAM-ACACTAGCCATCCTTACTGCGCTTCG-BBQ 3′ (94). The results are presented as the ΔΔCT-value using 18 ribosomal RNA as internal control (95).

### Virus titration

Supernatants from cells that had been infected with LACV wt or LACV ΔNS were collected and cleared by centrifugation at 800 × *g* for 5 minutes. The supernatants were titrated on Vero E6 with an overlay medium containing MEM (Gibco, 21935028) supplemented with 10% fetal bovine serum, 2 mM glutamine, 120 U/mL penicillin, 100 g/mL streptomycin (Gibco, 10378016), and 1,5% Avicel [FMC BioPolymer, (91)]. After 48 hours of incubation, the medium was removed and the cells washed with PBS before fixing with PBS-4% paraformaldehyde (Roth, 0335-4) for 24 hours at 4°C. Then, the fixed cells were again washed with PBS and permeabilized with PBS-0,1% Triton X-100 (Sigma, T9284-500ML) for 20 minutes. The cells were again washed and then incubated for 16 hours at 4°C with anti-LACV N (1:1,000, kind gift from Georg Kochs, University or Freiburg, Germany) in TBS-T buffer containing 1% non-fat milk powder. After washing the cells with PBS, they were incubated with secondary antibody IRDye800 conjugated anti-rabbit (1:10,000, Rockland, 611-132-122) and DRAQ5 (1:10,000, eBioscience, 65-0880-92) in TBS-T-1% milk powder at room temperature. Finally, the wells were washed first with PBS and then with H_2_O, and the fluorescence signals were detected and the foci counted using the Odyssey instrument (LI-COR).

### cDNA cloning

Primers for cloning human, *M. daubentonii* and *R. aegyptiacus* RIG-I, were generated using the In-Fusion cloning tool (https://www.takarabio.com/learning-centers/cloning/primer-design-and-other-tools) and ordered from Eurofins Genomics. The full-length sequences of RIG-I (DDX58) from *M. daubentonii* was assembled from previous data (29) while the human and *R. aegyptiacus* RIG-I sequences were available as GenBank entries (NM_014314.4 and XM_016130339.2, respectively). Primers were designed for cloning the cDNAs into the vector pI.18 with a 5′ 3×Flag-tag sequence added to the forward primer. Human RIG-I fwd: 5′ TGA CAC GAT CGG ATC CAT GGA CTA CAA AGA CCA TGA CGG TGA TTA TAA AGA TCA TGA TAT CGA TTA CAA GGA TGA CGA TGA CAA GAC CAC CGA GCA GCG ACG C 3′ and rev 5′ TCT AGA ATT CCT CGA GTC ATT TGG ACA TTT CTG CTG GA 3′; *M. daubentonii* and *R. aegyptiacus* RIG-I: fwd 5′ TGA CAC GAT CGG ATC CAT GGA CTA CAA AGA CCA TGA CGG TGA TTA TAA AGA TCA TGA TAT CGA TTA CAA GGA TGA CGA TGA CAA GAC GGC CGA GGA GCG GCG G 3′, *M. daubentonii* rev 5′ TCT AGA ATT CCT CGA GTC ATT TGG ACA TTT CTG CTG GAT C 3′, *R. aegyptiacus* rev 5′ TCT AGA ATT CCT CGA GTC ATT TGG GCA TTT CTG CAA CAT CG 3′. cDNAs were generated from RNAs isolated from A549, MyDauNi, and Ro6E-J cells that were treated for 16 hours with 1,000 U/mL IFN-α (B/D) (PBL Assay Science). The RNAs were isolated using RNeasy Mini Kit (Qiagen, Cat No./ID: 74106), and 1 µg was used for cDNA synthesis with the PrimeScript High Fidelity RT-PCR Kit (Takara, R022B). Of the cDNA, 2 µL were used as PCR template for amplification with the KOD Polymerase (Calbiochem, 71086-3). The PCR products were cloned into the pI.18 vector, digested with *Bam*HI (NEB, R3136S) and *Kpn*I (NEB, R3142S) using the In-Fusion Kit (Takara, 638911). The ligated product was transformed into Stellar competent cells accompanying the In-Fusion Kit, and spread onto agar plates at 37°C for 16 hours before colonies were picked for DNA isolation. Correctness of the inserts was confirmed by sequencing.

### Bioinformatics analysis

The full-length sequences of the cloned RIG-Is for *Homo sapiens*, *M. daubentonii*, and *R. aegyptiacus* were aligned to the respective published sequence for human (NM_014314.4) and *R. aegyptiacus* (XM_016130339.2) RIG-I, respectively, while the *M. daubentonii* sequence was compared to our assembled sequence from our previous study (29) (transcriptome available at https://osf.io/x9kad). The respective bat RIG-Is were completely conserved while the human RIG-I had a silent mutation at position 2709 (A to G) and 2760 (A to T). The DNA sequences were then *in silico* translated using the Expasy translation tool (https://web.expasy.org/translate/) and the resulting amino acid sequences aligned using T-Coffee (http://tcoffee.crg.cat/apps/tcoffee/do:regular) (96) and visualized with Boxshade (https://junli.netlify.app/apps/boxshade/; 3-fold alignment) or JalView (https://www.jalview.org; 17-fold alignment)(97). The respective domains were manually assigned based on Kolakofsky et al. (98) and confirmed by running the respective amino acid sequence in the SMART database (http://smart.embl-heidelberg.de/).

### Generation of ACE2-HEK293 ΔRIG-I cells

For efficient infection studies with SARS-CoV-2, we transduced HEK293 ΔRIG-I cells with a lentivirus expressing human ACE2 (hACE2, EC:3.4.17.23) under the control of an EF-1α promoter. For this purpose, we used the lentiviral expression system ViraPower (Thermofisher, K4975-00) and the transgene packaging vector pEGIP-Puro (pEGIP was a gift from Linzhao Cheng, Addgene Plasmid 26777; http://n2t.net/addgene:26777), which couples the expression of the transgene to the expression of the selection marker using an intra ribosomal entry site (IRES) (99). The pEGIP-derived packaging vector pEGIP-hACE2 was generated by homologous recombination using the NEBuilder Kit (NEB, E5520). The vector backbone was amplified using oligonucleotides with appropriate hACE2 sequence overhangs, namely pEGIP_hACE2_fwd 5′ TGATGATGTTCAGACCTCCTTTAGCCGCCCCCCCCCTCTC 3′ and pEGIP_hACE2_rev 5′ GCCAGGAAGAGCTTGACATCGATATCAAGCTTACCTAGC 3′. The hACE2 gene was amplified using the oligonucleotides ACE2_fwd 5′ ATGTCAAGCTCTTCCTGGCTC 3′ and ACE2_rev 5′ CTAAAAGGAGGTCTGAACATCATC 3′ from a plasmid containing the hACE2 mRNA. VSV-G pseudotyped lentiviral vector particles were produced in 293T cells by transfection of 1 µg pEGIP-hACE2 (transgene packaging vector), 0.8 µg pLP1 (gag, pol, and rev expression), 0.6 µg pLP2 (rev expression), and 0.3 µg pLP3 (VSV-G expression). Three days after transfection, the supernatant was harvested, sterile filtered through a 0.2 µm syringe adapter, and used to transduce 6 × 106 HEK293 ΔRIG-I cells. Two days after transduction, the supernatant was removed, cells were harvested by trypsinization and re-seeded in tenfold dilution series in DMEM containing 1 µg/mL puromycin. Single grown cell foci were scraped out of the cell culture dish after one week, cells were then separated by trypsinization and further selected by limited 10-fold dilution, yielding single cellular clones. After verification of hACE2 expression by immunofluorescence assays, the clonal cell line HEK293 ACE2 ΔRIG-I was expanded from one clone, cryo-preserved, and used for the experiments.

### RIG-I transcomplementation assays

A total of 5 × 10^4^ HEK293 ΔRIG-I, HEK293 ΔMAVS, ACE2-HEK293 ΔRIG-I, or MEF RIG-I^−/−^ cells were seeded in 24-well plates. After 24 hours, the cells were transfected with 0.25 µg of either pI.18-3×Flag-ΔMx (negative control), pI.18-3×Flag-huRIG-I, pI.18-3×Flag-myRIG-I, or pI.18-3×Flag-roRIG-I together with transfection control pLR-SV40-*Renilla* (Promega) and either of the reporter plasmids ISG54-Luc (100), p125-Luc (101), or pGL4.10 Rousettus IFN-ßp (102), as indicated in the respective figure, using the GeneJammer (Agilent, 204130) transfection reagent. Half of the plasmid transfected wells were stimulated by either transfecting 250 ng/well genomic VSV RNA or RVFV clone 13 RNA, using the Endofectin (GeneCopoeia, EF013) transfection reagent, or infecting the cells with RVFV clone 13 (MOI 10), as indicated in the respective figure. After 16 hours of incubation, the cells were lysed in Passive Lysis Buffer (Promega) and the firefly and *Renilla* luciferase activity measured using the Dual Luciferase Assay Kit (Promega, E1960) and a TriStar^2^ Multimode Reader LB942 (Berthold technologies). The data are presented as fold over unstimulated pI.18-3×Flag-ΔMx (CTRL) with firefly reporter values normalized to *Renilla* reporter control.

### Assay for RNA dependence of RIG-I

A total of 125 ng genomic RVFV clone 13 RNA was either mock treated or incubated with 40 U CIAP (Promega, M1821), 2 U RNaseIII (Ambion, AM2290), or 2 µg RNaseA (Ambion, AM2269) for 16 hours at 37°C. An aliquot of 25 ng/well of the treated RNAs were the mixed with either pI.18-3×Flag-ΔMx (CTRL), pI.18-3×Flag-huRIG-I, pI.18-3×Flag-myRIG-I, or pI.18-3×Flag-roRIG-I (25 ng/well each) together with transfection control pLR-SV40-*Renilla* (Promega) (50 ng/well) and the reporter plasmids ISG54-Luc (250 ng/well) (100) and transfected into HEK293 ΔRIG-I cells that were grown overnight in 24-well plates as described for the transcomplementation assay, using Endofectin (GeneCopoeia, EF013). After an additional 24 hours of incubation, firefly and *Renilla* luciferase activities were measured and processed as described for the transcomplementation assay.

### Isolation of genomic VSV and RVFV clone 13 RNA

Genomic RNAs were isolated from VSV or RVFV clone 13 particles by PEG800-precipitation and phenol-chloroform extraction as described (72). Briefly, the supernatant from a T175 flask infected with VSV or RVFV clone 13 (MOI 0.001) was collected and cleared by centrifugation at 800 × *g* for 5 minutes, after 48 hours (VSV), or 72 hours (RVFV) of incubation. The cleared supernatant was then mixed with 10.8 mL PEG8000 buffer (30% (wt/vol) PEG 8000, 10 mM Tris-HCl (pH 7.5), 1 mM EDTA, 100 mM NaCl). and 1.5 mL 5M NaCl followed by incubated for 1 hour at 4°C with head-over-tail rotation. The solution was then centrifugated at 4,600 × *g* for 1 hur at 4°C and the resulting pellet dissolved in 1.5 mL peqGOLD TriFast (peqlab, 30-2030). After vortexing and incubation at room temperature for 5 minutes, a phase-separation was performed by centrifugation at 4,600 × *g* for 11 minutes. The upper aqueous phase was collected and mixed with 700 µL chloroform (Roth, 7331) and glycogen (Roche, 10 901 393 001). The RNA was then precipitated at −20°C for 16 hours and pelleted by centrifugation at 4,600 × *g* for 33 minutes at 4°C. The pellet was washed twice with 70% Ethanol (Roth, 9065.4) and then dissolved in H_2_O to a final concentration of 250 ng/µL.

### Immunoblot analysis

Cell lysates were mixed 4:1 with 4 × sample buffer (143 mM Tris-HCl (pH 6,8), 28,6% Glycerol, 5,7% SDS, 4.3 mM Bromphenol Blue and 5% 2-mercaptoethanol) supplemented with phosphatase (Calbiochem, 524625) and protease (Roche, 04 693 116 001) inhibitors. The lysates were boiled for 10 minutes, separated by SDS-PAGE, and then blotted onto polyvinylidene difluoride membranes. The membranes were blocked with 5% (wt/vol) non-fat dry milk powder in TBS-T and probed with primary antibodies against the following targets: Flag (Sigma, F3165, 1:2,000, mouse, monoclonal), anti-β-tubulin (Abcam, ab6046, 1:1,000, rabbit, polyclonal), MAVS (Alexis, ALX-210-929, 1:1,000, rabbit, polyclonal), RVFV N (1:2,000, rabbit, polyclonal (serum), kind gift from Alejandro Brun), MDA5 (CellSignaling, 5321S, 1:1,000, rabbit, monoclonal), and SARS-CoV-2 NP (biomol, 200-401-A50, 1:2,000, rabbit, polyclonal). The secondary antibodies were peroxidase-conjugated antimouse IgG (Thermo Fisher, catalog no. 31430, 1:40,000, goat, polyclonal) and peroxidase-conjugated antirabbit IgG (Thermo Fisher, catalog no. 31460, 1:40,000, goat, polyclonal). Western blot signals were detected on a Chemidoc (Bio-Rad) using SuperSignal West Femto maximum sensitivity substrate (Thermo Scientific, catalog no. 34096).

### Poly I:C pull down

A total of 2 × 10^5^ HEK293 ΔRIG-I cells per well were seeded in 6-well plates. After 24 hours, the cells were transfected with 100 ng/well of either pI.18-3×Flag-ΔMx (CTRL), pI.18-3×Flag-huRIG-I, pI.18-3×Flag-myRIG-I, or pI.18-3×Flag-roRIG-I using GeneJammer (Agilent, 204130) transfection reagent. After 16 hours of incubation, the cells were scraped off in PBS and pelleted by centrifugation at 800 × *g* for 5 minutes. The cells were then lysed in lysis buffer [0.5% Triton X-100 (Sigma, T9284-500ML), 1× Protease inhibitor (c0mplete, Roche, 4693116001) in PBS] for 10 minutes at 4°C. Cell debris were removed by centrifugation at 15000 × *g*, 10 minutes, 4°C and the supernatants used for pull down. As input from each reaction, 5% of the volume was put aside. Dynabeads M-270 Streptavidin (Invitrogen, 65305) (250 µg/IP) was coupled to 100 ng/IP Poly(I:C) (HMW) Biotin (Invivogen) according to manufacturer’s instructions. After washing, the beads were re-suspended in the cell lysates and incubated at 4°C with head over tail rotation for 16 hours. To compete for RIG-I binding, 10 µg of VSV RNA was added to the lysate before incubation. After incubation the beads were washed three times in lysis buffer and the bound fraction eluted in 0.5% Triton X-100 in PBS mixed 4:1 in 4 × sample buffer supplemented with phosphatase and protease inhibitors. The samples were then boiled and the supernatant used for immunoblot analysis.

### SARS-CoV-2 infection

HEK293 ΔRIG-I-ACE2 cells, 5 × 10^4^ cells/well, were seeded in 24-well plates treated with Poly-D-lysine hydrobromide (Sigma, P6407-5MG) and reverse transfected with 250 ng/well of either pI.18-3×Flag-ΔMx, pI.18-3×Flag-huRIG-I, pI.18-3×Flag-myRIG-I or pI.18-3×Flag-roRIG-I using GeneJammer transfection reagent (Agilent technologies, 204131). After 24 hours of incubation, the cells were infected with SARS-CoV-2 or RVFV clone 13 at an MOI of 1 under BSL3 conditions. After 16 hours of incubation, the medium was removed, the cells washed with PBS, and the samples taken for RT-qPCR or immunoblotting.

### siRNA treatment

ACE2-HEK293 ΔRIG-I cells were seeded at 5 × 10E4 cells per well in 24-well plates that had been treated with Poly-D-lysine hydrobromide (Sigma, P6407-5MG) and reverse transfected with 50 nM/well of control (CTRL) or MDA5 siRNA (Qiagen, FlexiTube Genesolution Cat. No.: 1027280 and 1027416) using Lipofectamine RNAiMAX (Thermo Fisher, 13778075). After 4 hours of incubation, the medium was exchanged and the cells transfected with 250 ng/well of either pI.18-3×Flag-ΔMx, pI.18-3×Flag-huRIG-I, pI.18-3×Flag-myRIG-I, or pI.18-3×Flag-roRIG-I using GeneJammer transfection reagent (Agilent technologies, 204131). After 24 hours of incubation, the cells were infected with SARS-CoV-2 or RVFV clone 13 at an MOI of 1 as described above. After 16 hours of incubation, the medium was removed, the cells washed with PBS, and the samples taken for RT-qPCR or immunoblotting.

### Statistical analysis

Two-tailed paired *t* tests were done using GraphPad Prism (version 7.05, GraphPad Software Boston, USA) based on the logarithmic values for viral growth assays while all other comparisons were made on fold-induction over control values.

## SUPPLEMENTAL MATERIAL

Supplemental figures jvi.00205-23-s0001.pdf Supplemental Figures S1 to S6 including legends.

ASM does not own the copyrights to Supplemental Material that may be linked to, or accessed through, an article. The authors have granted ASM a non-exclusive, world-wide license to publish the Supplemental Material files. Please contact the corresponding author directly for reuse.
