# Supplementary material for: Functional comparisons of the virus sensor RIG-I from humans, the microbat Myotis daubentonii, and the megabat Rousettus aegyptiacus, and their response to SARS-CoV-2 infection
Source: J Virol. 2023 Sep 20;97(10):e00205-23. doi: 10.1128/jvi.00205-23 (PMC10653997; doi:10.1128/jvi.00205-23)
Supplement: Supplemental figures — S1 to S6 including legends. [file jvi.00205-23-s0001.pdf]

Suppl. Figure S1

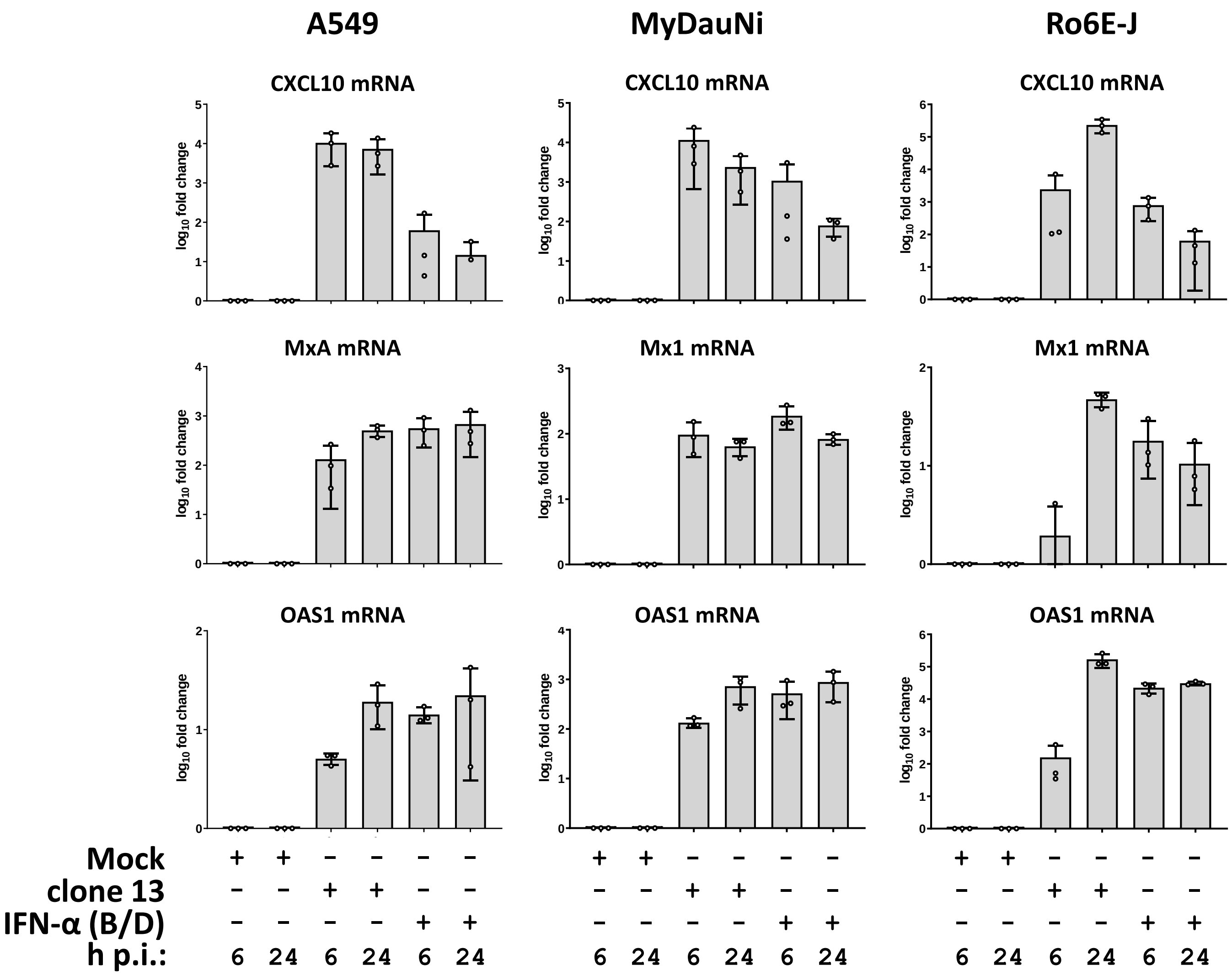

Suppl. Figure S1. Continuation of Fig. 1A to show type I Interferon competence of micro- and megabat cells lines. cDNAs from cells described in figure 1 were analysed for mRNA expression of CXCL10, MxA/Mx1 and OAS1 by RT-qPCR of the respective species. The graphs show the fold induction over mock data points, with mean values and standard deviations from three independent replicates.

Suppl. Figure S2

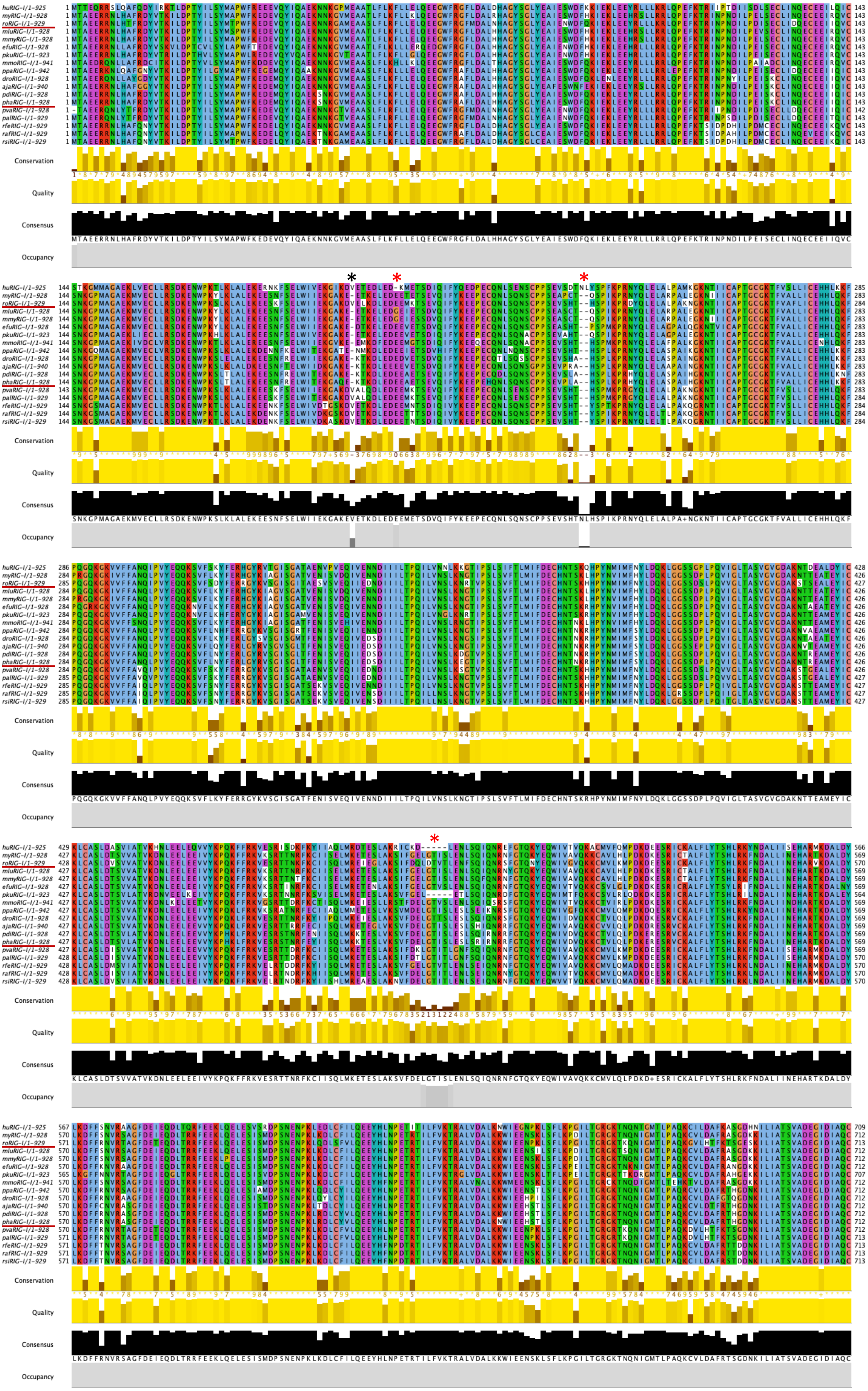

Suppl. Figure S2 Cont.

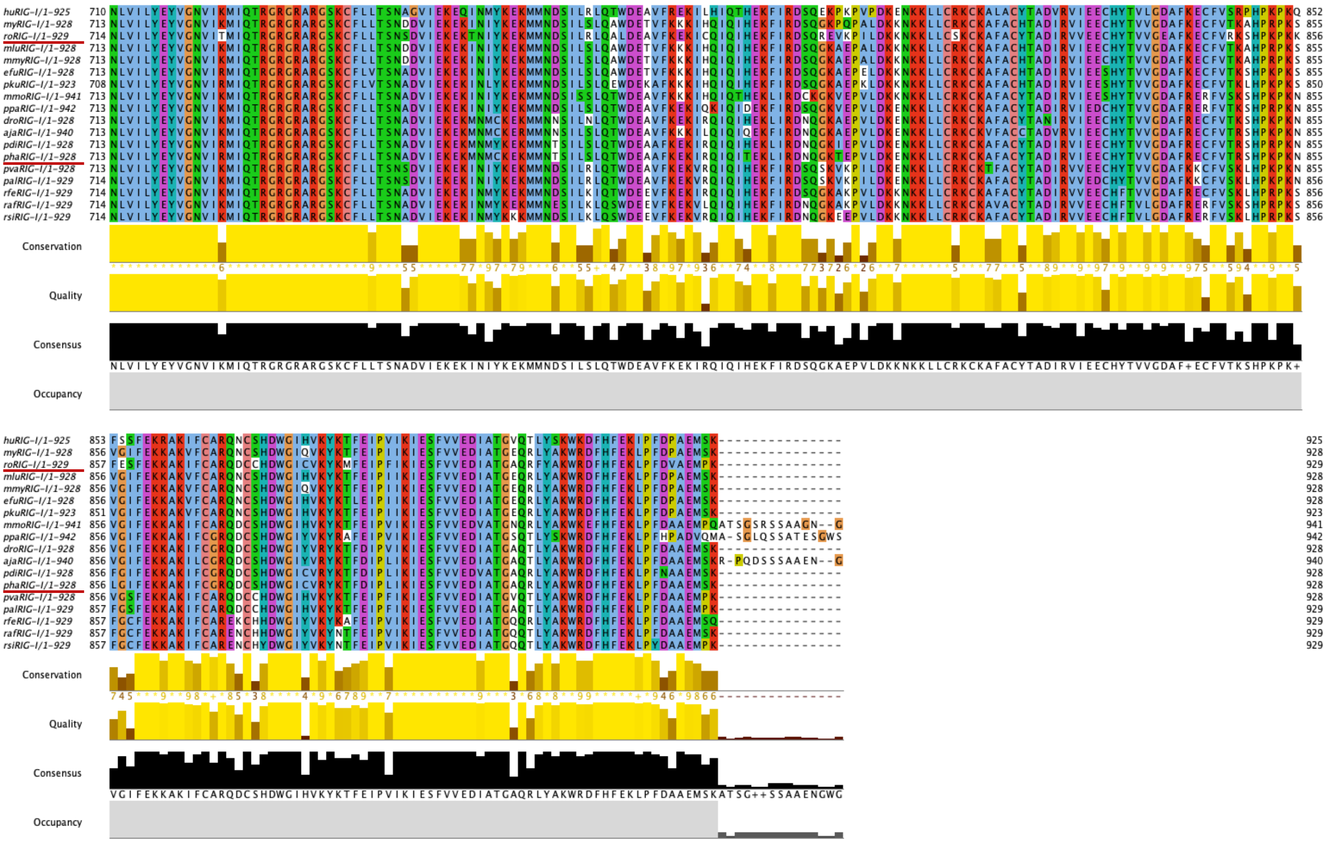

Suppl. Figure S2. Extended amino acid sequence alignment for human, micro- and megabat RIG-I. Multiple amino acid sequence alignments using the huRIG-I, myRIG-I, and roRIG-I sequences from figure 2 and additional sequences from the *Yangochiroptera* members *Myotis lucifugus* (mluRIG-I; XP_014319864.1), *Myotis myotis* (mmyRIG-I; KAF6314384.1), *Eptesicus fuscus* (pkuRIG-I; XP_008140227.2), *Pipistrellus kuhlii* (pkuRIG-I; KAF6331617.1), *Molossus molossus* (mmoRIG-I; XP_036117415.1), *Pteronotus parnellii mesoamericanus* (ppaRIG-I; XP_054438908.1), *Desmodus rotundus* (droRIG-I; ATW00980.1), *Artibeus jamaicensis* (ajaRIG-I; XP_037015789.2), *Phyllostomus discolor* (pdiRIG-I; XP_028385969.1), *Phyllostomus hastatus* (phaRIG-I; XP_045696202.1), and the *Yinpterochiroptera* members *Pteropus vampyrus* (pvaRIG-I; ENSPVAP00000008684), *Pteropus alecto* (palRIG-I; ELK09090.1), *Rhinolophus ferrumequinum* (rfeRIG-I; XP_032979882.1), *Rhinolophus affinis* (rafRIG-I; AJO25033.1), *Rhinolophus sinicus* (rsiRIG-I; AJO25032.1). Red lines separate the three RIG-Is that were functionally compared in this study from additional sequences of *Yangochiroptera* and *Yinpterochiroptera* members. The red asterisks (*) are highlighting the indels that distinguish human and bat RIG-I sequences, and the black asterisk marks the *Yangochiroptera*-specific deletion (see Fig. 2 and text).

Suppl. Figure S3

huRIG-I

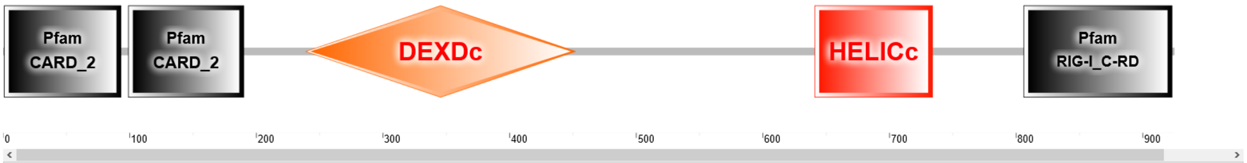

Information | Architecture | Interactions | PTMs | Orthology

**Length**	925 aa
**Source database**	UniProt
**Identifiers**	DDX58_HUMAN, O95786, ENSP00000369213.2, ENSP00000369213, A2RU81, Q5HYE1, Q5VYT1, Q9NT04, H2QX44_PANTR, H2QX44, A2A376_HUMAN, A2A376, A0A2J8QSR6_PANTR, A0A2J8QSR6
**Source gene**	ENSG00000107201
**Alternative splicing**	ENSP00000369197.1, DDX58_HUMAN

The SMART diagram above represents a summary of the results shown below. Domains with scores less significant than established cutoffs are not shown in the diagram. Features are also not shown when two or more occupy the same piece of sequence; the priority for display is given by **SMART > PFAM > PROSPERO repeats > Signal peptide > Transmembrane > Coiled coil > Unstructured regions > Low complexity**. In either case, features not shown in the above diagram are marked as '**overlap**' in the right side table below.

Confidently predicted domains, repeats, motifs and features:

Name	Start ▲	End	E-value
Pfam:CARD_2	1	93	1.3e-30
Pfam:CARD_2	99	190	2.2e-26
DEXDc	239	452	1.57e-28
AAA	256	440	0.0093
HELICc	641	734	4.59e-12
Pfam:RIG-I_C-RD	806	923	6.1e-40

Click on a row to highlight the feature in the diagram above. Click the feature name for more information.

Features NOT shown in the diagram:

Name	Start ▲	End	E-value	Reason
low complexity	56	67	N/A	overlap
low complexity	95	110	N/A	overlap
Pfam:ResIII	234	413	8.5e-18	overlap
Pfam:DEAD	244	420	7.5e-18	overlap
Pfam:Helicase_C	609	734	1.1e-15	overlap
low complexity	806	818	N/A	overlap

Click on a row to highlight the feature in the diagram above. Click the feature name for more information.

myRIG-I

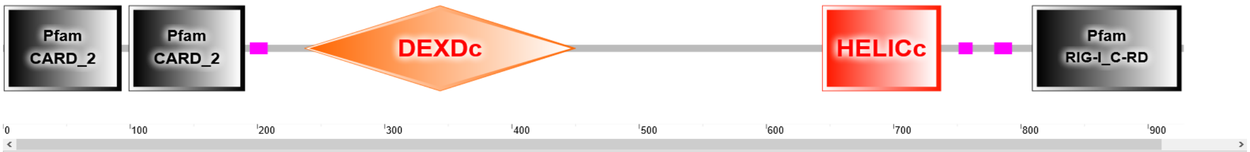

Architecture

Domain architecture analysis

Display all proteins with similar:

- Domain organisation: Proteins having all the domains as the query in the same order. Additional domains are allowed.
- Domain composition: Proteins with the same domain composition have at least one copy of each of domains of the query.

The SMART diagram above represents a summary of the results shown below. Domains with scores less significant than established cutoffs are not shown in the diagram. Features are also not shown when two or more occupy the same piece of sequence; the priority for display is given by **SMART > PFAM > PROSPERO repeats > Signal peptide > Transmembrane > Coiled coil > Unstructured regions > Low complexity**. In either case, features not shown in the above diagram are marked as '**overlap**' in the right side table below.

Confidently predicted domains, repeats, motifs and features:

Name	Start ▲	End	E-value
Pfam:CARD_2	1	93	4.7e-27
Pfam:CARD_2	99	190	2.4e-27
low complexity	194	208	N/A
DEXDc	237	450	3.61e-28
HELICc	644	737	1.15e-12
low complexity	751	762	N/A
low complexity	779	793	N/A
Pfam:RIG-I_C-RD	809	926	2.5e-36

Click on a row to highlight the feature in the diagram above. Click the feature name for more information.

Features NOT shown in the diagram:

Name	Start ▲	End	E-value	Reason
Drf_GBD	1	144	140000	threshold
RQC	11	119	49400	threshold
Bet_v_1	51	206	68100	threshold
low complexity	56	64	N/A	overlap
RGS	57	180	1620	threshold
ENT	58	122	168000	threshold
DSHCT	66	165	64200	threshold
DUF4206	78	177	38200	threshold
Cullin_Nedd8	89	147	118000	threshold

roRIG-I

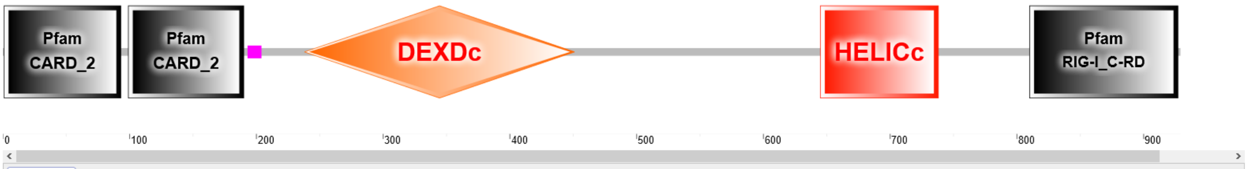

Architecture

Domain architecture analysis

Display all proteins with similar:

- Domain organisation: Proteins having all the domains as the query in the same order. Additional domains are allowed.
- Domain composition: Proteins with the same domain composition have at least one copy of each of domains of the query.

The SMART diagram above represents a summary of the results shown below. Domains with scores less significant than established cutoffs are not shown in the diagram. Features are also not shown when two or more occupy the same piece of sequence; the priority for display is given by **SMART > PFAM > PROSPERO repeats > Signal peptide > Transmembrane > Coiled coil > Unstructured regions > Low complexity**. In either case, features not shown in the above diagram are marked as '**overlap**' in the right side table below.

Confidently predicted domains, repeats, motifs and features:

Name	Start ▲	End	E-value
Pfam:CARD_2	1	93	3.8e-27
Pfam:CARD_2	99	190	1.1e-25
low complexity	193	204	N/A
DEXDc	238	451	2.09e-24
HELICc	645	738	1.32e-12
Pfam:RIG-I_C-RD	810	927	2.8e-34

Click on a row to highlight the feature in the diagram above. Click the feature name for more information.

Features NOT shown in the diagram:

Name	Start ▲	End	E-value	Reason
PWI	4	67	1500	threshold
BP28CT	6	182	122000	threshold
zf-PARP	52	102	64800	threshold
Pfam:Latarcin	55	120	0.78	threshold
BING4CT	56	120	55200	threshold
low complexity	56	67	N/A	overlap
RGS	57	180	1500	threshold

Suppl. Figure S3. SMART result for human, micro- and megabat RIG-I. The amino acid sequences for our cloned human and bat RIG-I orthologs were analyzed using the SMART database. Pictures of the output are shown.

Suppl. Figure S4

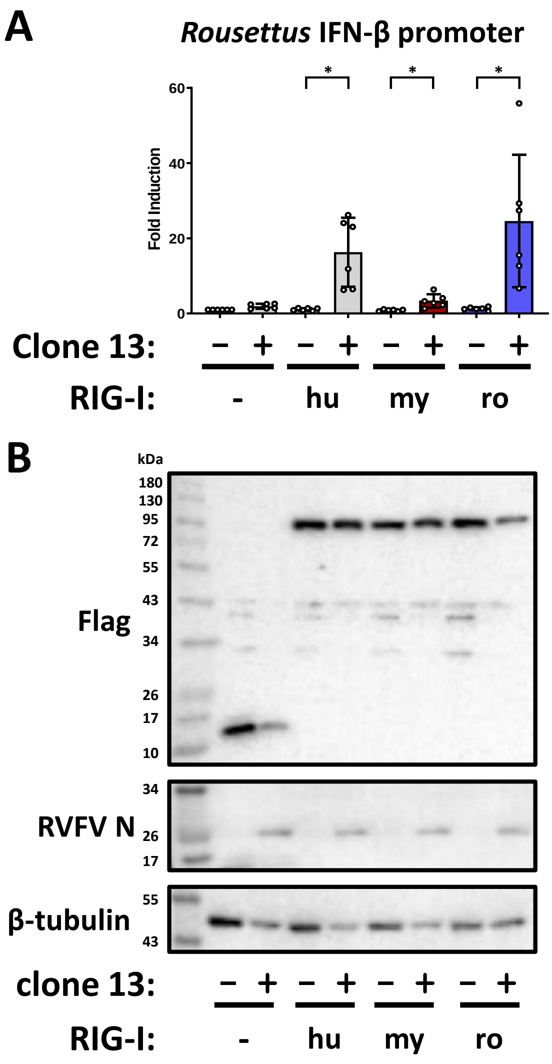

Suppl. Figure S4. Transcomplementation of RIG-I-deficient mouse cells by bat RIG-I orthologs. A) MEF RIG-I$^{-/-}$ cells were transfected with plasmids encoding CTRL, hu-, my- or roRIG-I together with firefly-luciferase expressing plasmids under the control of the *Rousettus* IFN-β promoter and *Renilla*-luciferase under SV40-control promoter. After 24 h the cells were stimulated by infection with RVFV clone 13 (MOI 10) and 16 h later analysed for firefly/*Renilla* luciferase activities. The graphs show the fold induction over CTRL data points, with mean values and standard deviations from three independent replicates. **B)** Immunoblot analysis with antibodies against the indicated antigens. Representative data from three independent experiments are shown. * $p<0,05$.

Suppl. Figure S5

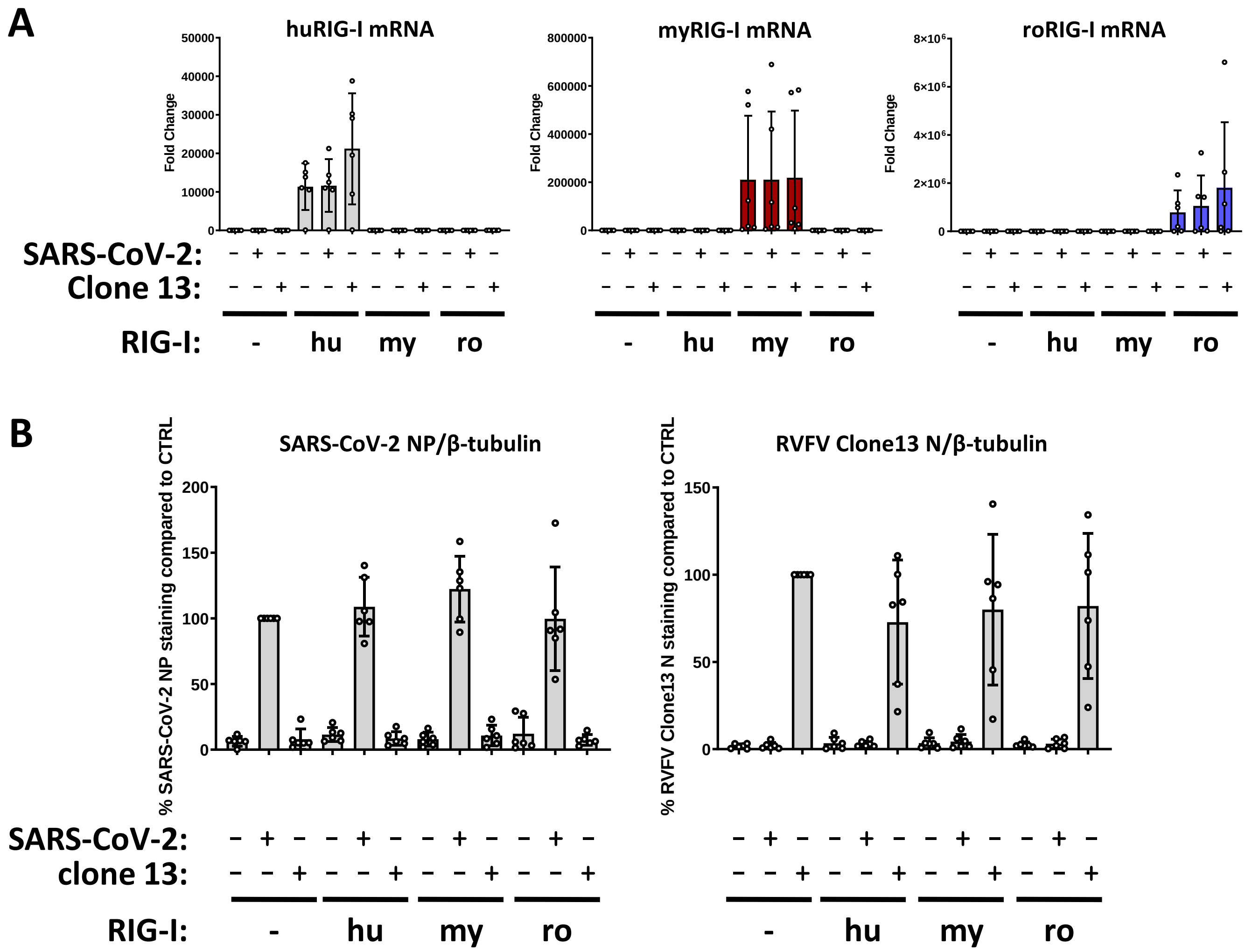

Suppl. Figure S5. Extended control panel for figure 7. A) cDNAs from cells described in figure 7A were analysed by RT-qPCR for mRNA expression of RIG-I of the respective species. The graphs show the fold induction over mock data points, with mean values and standard deviations from three independent replicates. **B)** Quantification of the immunoblot analyses for viral nucleoproteins, normalized to ß-tubulin, as shown in figure 7B.

Suppl. Figure S6

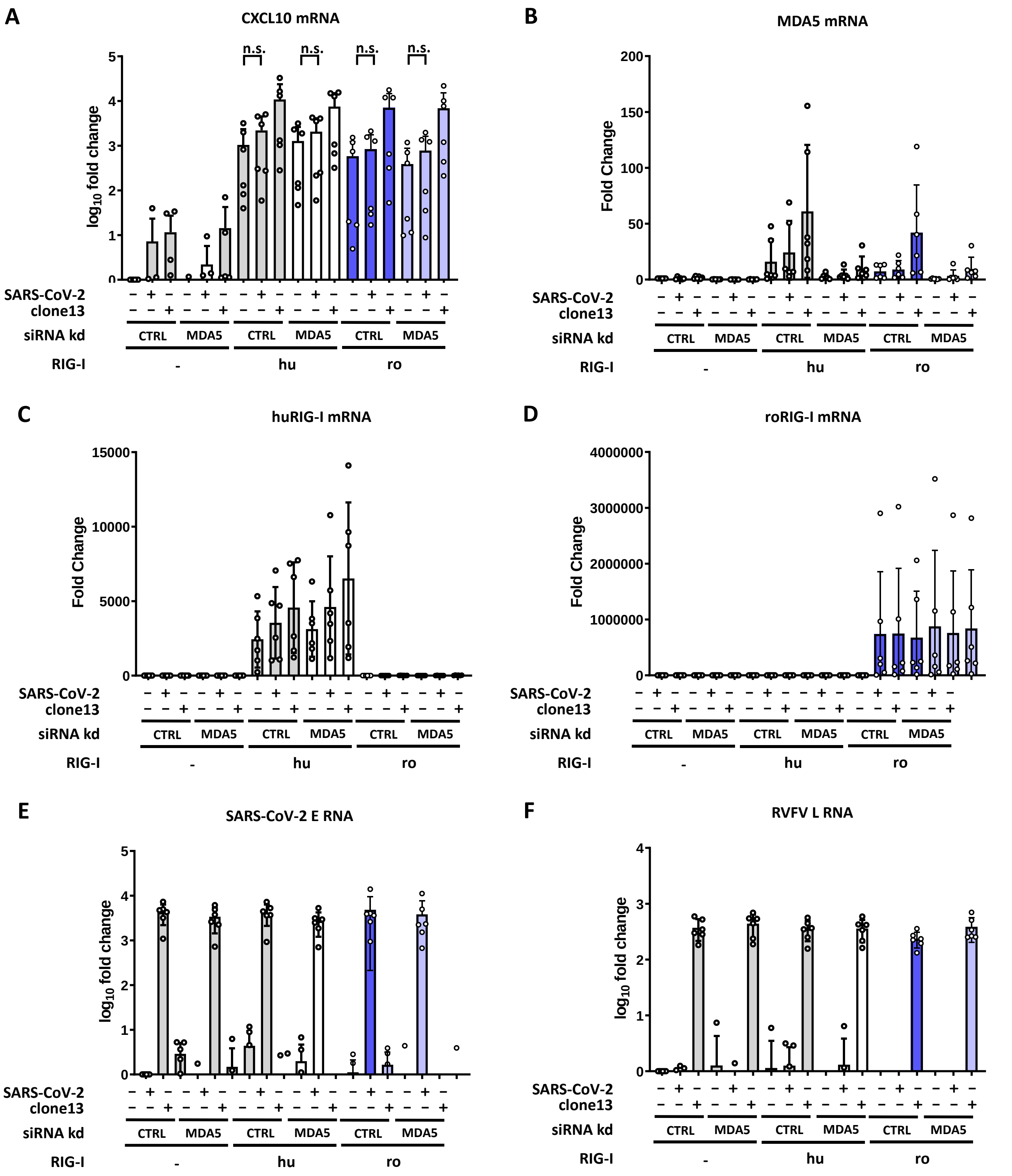

Suppl. Figure S6. Extended control panel for figure 8. A) to **F)** cDNAs from cells described in figure 8 were analysed by RT-qPCR for the presence of the indicated RNA sequences. n.s.: non-significant, * p<0.05.
